# Insulin and obesity transform hypothalamic-pituitary-adrenal axis stemness and function in a hyperactive state

**DOI:** 10.1016/j.molmet.2020.101112

**Published:** 2020-11-04

**Authors:** Martin Werdermann, Ilona Berger, Laura D. Scriba, Alice Santambrogio, Pia Schlinkert, Heike Brendel, Henning Morawietz, Andreas Schedl, Mirko Peitzsch, Aileen J.F. King, Cynthia L. Andoniadou, Stefan R. Bornstein, Charlotte Steenblock

**Affiliations:** 1 Department of Internal Medicine III, University Hospital Carl Gustav Carus, Dresden University of Technology, Fetscherstraße 74, Dresden, 01307, Germany; 2 Centre for Craniofacial and Regenerative Biology, King's College London, Guy's Hospital, London, SE1 9RT, UK; 3 Department of Pharmacology and Toxicology, University Hospital Carl Gustav Carus, Dresden University of Technology, Fetscherstraße 74, Dresden, 01307, Germany; 4 Division of Vascular Endothelium and Microcirculation, Department of Medicine III, University Hospital Carl Gustav Carus Dresden, Dresden University of Technology, Fetscherstraße 74, Dresden, 01307, Germany; 5 University of Côte d'Azur, INSERM, CNRS, iBV, Parc Valrose, Nice, 06108, France; 6 Institute of Clinical Chemistry and Laboratory Medicine, University Hospital Carl Gustav Carus, Dresden University of Technology, Fetscherstraße 74, Dresden, 01307, Germany; 7 Department of Diabetes, School of Life Course Sciences, King's College London, Great Maze Pond, London, SE1 9RT, UK; 8 Diabetes and Nutritional Sciences Division, King's College London, Guy's Campus, London, SE1 1UL, UK

**Keywords:** Adrenal, Pituitary, HPA axis, Progenitors, Obesity, Metabolic stress, ACTH, adrenocorticotropic hormone, AgRP, agouti-related peptide, bFGF, basic fibroblast growth factor, CRH, corticotropin-releasing hormone, DMEM, Dulbecco's modified Eagle medium, FACS, fluorescence-activated cell sorting, HBSS, Hank's Balanced Salt Solution, HFD, high-fat diet, HPA, hypothalamic-pituitary-adrenal, ND, normal diet, NPY, neuropeptide Y, PBS, phosphate-buffered saline, PI, propidium iodide, T2D, type 2 diabetes, zF, zona fasciculata, zG, zona glomerulosa

## Abstract

**Objective:**

Metabolic diseases are an increasing problem in society with the brain-metabolic axis as a master regulator of the human body for sustaining homeostasis under metabolic stress. However, metabolic inflammation and disease will trigger sustained activation of the hypothalamic-pituitary-adrenal axis. In this study, we investigated the role of metabolic stress on progenitor cells in the hypothalamic-pituitary-adrenal axis.

**Methods:**

*In vitro*, we applied insulin and leptin to murine progenitor cells isolated from the pituitary and adrenal cortex and examined the role of these hormones on proliferation and differentiation. *In vivo*, we investigated two different mouse models of metabolic disease, obesity in leptin-deficient *ob/ob* mice and obesity achieved via feeding with a high-fat diet.

**Results:**

Insulin was shown to lead to enhanced proliferation and differentiation of both pituitary and adrenocortical progenitors. No alterations in the progenitors were noted in our chronic metabolic stress models. However, hyperactivation of the hypothalamic-pituitary-adrenal axis was observed and the expression of the appetite-regulating genes *Npy* and *Agrp* changed in both the hypothalamus and adrenal.

**Conclusions:**

It is well-known that chronic stress and stress hormones such as glucocorticoids can induce metabolic changes including obesity and diabetes. In this article, we show for the first time that this might be based on an early sensitization of stem cells of the hypothalamic-pituitary-adrenal axis. Thus, pituitary and adrenal progenitor cells exposed to high levels of insulin are metabolically primed to a hyper-functional state leading to enhanced hormone production. Likewise, obese animals exhibit a hyperactive hypothalamic-pituitary-adrenal axis leading to adrenal hyperplasia. This might explain how stress in early life can increase the risk for developing metabolic syndrome in adulthood.

## Highlights

• Insulin enhances proliferation and differentiation of adrenocortical and pituitary progenitors.
• Obesity leads to hyperactivation and priming of the HPA axis.
• Obesity leads to overexpression of appetite-regulating genes in the hypothalamus.
• Obesity leads to a decrease in the expression of appetite-regulating genes in the adrenal gland.

## 1 Introduction

Metabolic diseases such as obesity, type 2 diabetes (T2D), or metabolic syndrome are major challenges in modern medicine, and psychological stress has been incriminated as a contributing factor [1,2]. Dysregulation of the sympatho-adrenomedullary system and frequent or chronic stimulation of the hypothalamic-pituitary-adrenal (HPA) axis have been implicated and may contribute to the current increase in metabolic disorders [3,4]. Dysfunction of the endocrine stress system characterized by sustained hyper- or hypoactivity leads to various pathological states. Some of these states show features common to metabolic syndrome such as hypertension, insulin resistance, and visceral obesity [5]. Furthermore, HPA axis activity and the release of steroid hormones can be modulated by factors regulating feeding behavior [[6], [7], [8]].

To regulate body weight, the hypothalamus must interpret and integrate incoming signals such as levels of glucose, free fatty acids, and amino acids but also hormones such as leptin and insulin from the adipose tissue and pancreas, respectively. The hypothalamus then synthesizes and secretes corticotropin-releasing hormone (CRH), which regulates pituitary adrenocorticotropic hormone (ACTH) secretion, leading to glucocorticoid release from the adrenal cortex [9]. In addition to this function, CRH in the arcuate nucleus in the hypothalamus inhibits neuropeptide Y (NPY) and agouti-related peptide (AgRP)-expressing neurons [10,11]. These cells stimulate feeding behavior and suppress energy expenditure, meaning that after acute stress, appetite is decreased [12,13]. However, chronic stress leads to a constant elevation of glucocorticoids in the bloodstream, which contributes to visceral fat accumulation and insulin resistance [14]. Furthermore, glucocorticoids act on the hypothalamus to stimulate appetite by upregulating NPY and AgRP [[15], [16], [17]]. Chronic stress contributes to an altered energy balance and feeding behavior, leading to an increased vulnerability to developing metabolic disorders [[18], [19], [20]].

Glucocorticoids also stimulate the release of leptin, an appetite-suppressing hormone, from adipose tissue. Glucocorticoids also reduce the brain's sensitivity to leptin, contributing to leptin resistance [21,22]. Conversely, excessive weight gain regulates the release of various factors, including cytokines, peptides, and neurotransmitters, which induce adrenal steroid secretion for proper adjustment to homeostatic challenges [6,23,24]. The adrenal and pituitary glands then undergo remodeling [25,26]. To date, many studies have provided evidence that epigenetic changes such as DNA methylation and histone modifications, both involving chromatin remodeling, contribute to fetal metabolic programming. Epigenetic changes in utero due to impaired supply of nutrients (under- or overnutrition) might lead to changes in physiology and metabolism in target tissues [27,28]. For example, epigenetic changes were observed in the genes *Pomc* (among others encoding ACTH), *Npy*, and *Lep* [29,30]. These epigenetic changes were also suggested to occur in stem cells [31,32]. Adult stem and progenitor cells exist in different components of the brain-metabolic axis and are suggested to play an important role in organ maintenance and plasticity in response to changing neural stimuli and physiological needs [[33], [34], [35], [36]]. However, the role of adult stem cells and progenitors in metabolic disease remains unknown. We recently identified and characterized a subpopulation of Nestin (+) adrenocortical progenitors. Under mental stress, centripetal migration and differentiation into steroidogenic cells are enhanced in these cells [37,38]. Additionally, in Nestin (+) cells in the hippocampus, oligodendrogenesis is increased under stress [39]. This suggests that Nestin (+) subpopulations in different tissues are remarkably capable of reacting to physiological needs [40,41]. Therefore, these cells are of specific interest in our studies.

In this investigation, we examined whether Nestin (+) cell populations can be stimulated by factors regulating feeding behavior and thereby contribute to altered HPA function in metabolic disease. We characterized the effects of metabolic factors on Nestin (+) cells in both the adrenal cortex and anterior lobe of the pituitary gland. These cells show progenitor characteristics and are stimulated by insulin *in vitro*. Steroidogenesis and ACTH production in cultures of Nestin (+) cells and their descendants are enhanced, strengthening the idea of cell populations being prone to metabolic stress. Moreover, we show that different types of stressors regulate hypothalamic and adrenal genes involved in energy balance, hinting at shared underlying adaptive stress response mechanisms. This may suggest a uniform and coordinated signature and programming within the entire endocrine stress axis.

## 2 Materials and methods

### 2.1 Animals

The animal research ethics committee of Dresden University of Technology and the Regional Council of Saxony (Landesdirektion Sachsen) approved the animal experiments according to institutional guidelines and German animal welfare regulations. Animal husbandry was carried out under compliance of the Animals (Scientific Procedures) Act 1986, Home Office license, and King's College London ethical review approval. Animal experiments using obese *ob/ob* mice (both male and female) were conducted in London, UK. All the other animal experiments were carried out in Dresden, Germany.

Heterozygous Nestin-GFP transgenic mice [42] with a C57BL/6 N genetic background were generated as described previously [43]. For lineage-tracing studies, Nes-CreERT mice [44] (Jackson Laboratory, stock 012,906) were bred with Rosa26-eYFP mice [45] also with a C57BL/6 N background. Eight-week-old C57BL/6NRj WT mice were obtained from Janvier Laboratory.

Female homozygous obese (*ob/ob*) and heterozygous lean (*ob/+*) mice were obtained from Envigo. Male *ob/ob* mice and control animals were obtained from Charles River. Control animals for male *ob/ob* mice were wild-type animals*.* Female and male *ob/ob* mice were generated with a C57Bl/6 J genetic background.

All the mouse colonies were maintained under a 12:12 h light/dark cycle and fed ad libitum. Unless otherwise stated, the mice were sacrificed by cervical dislocation.

### 2.2 Isolation and culture of adrenal cells

Adrenals of 10–15 mice (age 2–5 months, both sexes) per experiment were excised and placed in petri dishes with ice-cold PBS. Fat tissue surrounding the adrenals was carefully removed and the adrenal cortex was thoroughly isolated from the medulla. All the cortical tissues were pooled, pelleted (350×*g*, 5 min), and digested for 20 min at 37 °C while shaking (1.8 mg/ml of collagenase, 10 mg/ml of BSA, and 0.18 mg/ml of DNase in PBS, all from Sigma–Aldrich). The digestion was stopped by washing twice in PBS and the cells were resuspended in 1 ml of high-glucose Dulbecco's modied Eagle medium (DMEM/F12, Gibco, Thermo Fisher Scientific) containing 10% fetal bovine serum (FBS) (Biochrom), 1% antibiotic-antimycotic solution (Gibco, Thermo Fisher Scientific), 1% l-glutamine (PAA Laboratories), and 20 ng/ml of basic fibroblast growth factor (bFGF) (Sigma–Aldrich). Isolated cells were cultured in ultra-low-attachment surface plates (Corning) at 37 °C in a humidified atmosphere (95% O_2_, 5% CO_2_). To induce Cre recombination *in vitro* in cells isolated from the *Nes-CreERT*^*+/-*^*;Rosa26-eYFP*^+/+^ mice, 1 μM of 4OH-tamoxifen (Sigma–Aldrich) was added to the culture the first 3 days after isolation. Stimulation of the adrenocortical cells with 4 μg/ml of insulin (Thermo Fisher Scientific) or 1 μg/ml of leptin (Sigma–Aldrich) started on day 2 and continued throughout the entire experiment.

### 2.3 Differentiation of adrenocortical cells

To assess *in vitro* differentiation of isolated adrenal cells, spheroids (after 7 days of proliferation) were plated into 24-well plates (Corning) or 8-well chamber plates (ibidi) coated with 1 mg/ml of poly-d-lysine (Merck Millipore) and 3 μl/ml of fibronectin (R&D Systems) and cultured in the absence of bFGF. The medium was replaced with fresh medium every 2–3 days (containing 4 μg/ml of insulin or 1 μg/ml of leptin).

### 2.4 Isolation and culture of pituitary cells

Anterior pituitaries were mechanically dissociated into a single-cell suspension following incubation in enzyme mix (0.5% w/v collagenase [Sigma–Aldrich], 0.1 X of Trypsin–EDTA [Gibco], and 50 μg/ml of DNase [Sigma–Aldrich] in Hank's Balanced Salt Solution [HBSS, Gibco]) for 1.5–2 h at 37 °C with frequent agitation. After washing in HBSS, the cells were resuspended in pituitary stem cell medium (DMEM-F12 [Gibco] containing 5% fetal bovine serum [Merck], 20 ng/ml of bFGF [Sigma–Aldrich], and 50 ng/ml of cholera toxin [Sigma–Aldrich]). The medium containing 4 μg/ml of insulin or 1 μg/ml of leptin was replaced by fresh medium every 2–3 days. After seven days in culture, adherent colonies were either fixed and permeabilized with 0.5% Triton-X in PBS, washed with PBS, and stained using hematoxylin (SAV Liquid Production) for 3 min or used for RNA isolation or immunofluorescence. Images of plates stained with hematoxylin were captured using a gel documentation system (PeqLAB).

### 2.5 Fluorescent-activated cell sorting

After dissociation into a single-cell suspension following washes in HBSS, anterior pituitary cells were suspended in PBS supplemented with 1% fetal bovine serum, 25 mM of HEPES (Gibco), and 10 μg/μl of propidium iodide (PI) (BioLegend). For *in vitro* proliferation and differentiation of Nestin (+) pituitary cells alone, GFP(+) and GFP(−) cells from anterior pituitaries of the Nestin-GFP mice were flow sorted on a FACSAriaII flow cytometer (BD Biosciences) via FACSDiva software using PI as a live/dead cell marker (Fig. S1). The cells were sorted directly into pituitary stem cell medium. The cells were transferred to 6-well plates and cultured as previously described.

### 2.6 Immunofluorescence of cultured cells

Cultured cells were fixed in 4% PFA in PBS for 15 min. After washing in phosphate-buffered saline (PBS, Sigma–Aldrich), blocking buffer (PBS containing 1% BSA, 0.1% Triton-X, and 5% goat or donkey serum) was added for 1 h. Blocking buffer was replaced with primary antibody (Table S1) in PBS containing 1% BSA and 5% goat or donkey serum at 4 °C overnight. The next day, the cells were washed in PBS and secondary antibody (Table S1) diluted in PBS was added for 2 h at room temperature. After washing with PBS, nuclei were stained with 4ʹ-6-diamidino-2-phenylindole (DAPI, Thermo Fisher Scientific) and the cells were left in PBS for imaging. SOX2 antibodies were previously validated [33]. Other antibodies were validated as shown in Figs. S3 and S5.

### 2.7 Quantitative real-time PCR

Total RNA was isolated using a NucleoSpin RNA Plus XS (Macherey–Nagel) kit and reverse transcription was conducted using the Moloney MLV Reverse Transcription system (Promega). qRT-PCR was performed using a Light Cycler 1.5 System (Roche) or a CFX Connect Real-Time PCR Detection System (Bio-Rad) and a SYBR Green RT-PCR kit according to the manufacturer's instructions (Qiagen) for all the genes except *Cyp11b1* and *Cyp11b2*, which were measured using the Light Cycler Taq Man Master system (Roche Life Science). Primers used are shown in Table S2. qRT-PCR analyses were conducted in triplicate, and Ct values were normalized against the internal control gene *beta-a*ctin. Fold differences in expression levels were calculated according to the comparative Ct method [46].

### 2.8 Electron microscopy

Adrenocortical cells were cultured on ACLAR foil in 24-well plates and after differentiation, they were fixed in 2.5% glutaraldehyde in 0.1 M of phosphate buffer (PB) for 2 h. The cells were then washed in 1% osmium tetroxide in PB for 1 h. Dehydration in 50%, 70%, 90%, 96%, and 3 × 100% EtOH for 10 min each was performed. A mixture of 100% EtOH and Epon mixed 1:1 with propylene oxide was then added for 2 h. Pure Epon was added followed by polymerization for 48 h at 60 °C. For immunostaining, the cells were fixed in 4% paraformaldehyde in PB followed by dehydration as previously described and then embedded in LR White. The samples were then incubated with anti-GFP and subsequently with gold-labeled secondary antibody. Ultra-thin sections (70 nm) were stained with 2% uranyl acetate for 10 min and 0.4% lead citrate for 5 min and examined at 80 kV using a CM 10 electron microscope (Philips, Eindhoven, the Netherlands).

### 2.9 Steroid profiling by liquid chromatography-tandem mass spectrometry (LC-MS/MS)

The medium of *in vitro* cultured adrenal cells was changed and after 24 h a conditioned medium sample was collected. Steroid hormones (aldosterone and corticosterone) were measured by LC-MS/MS as previously described [47,48]. The steroid levels were quantified by comparing the ratios of analyte peak areas obtained from the samples to the respective peak areas of stable isotope-labeled internal standard calibrators.

### 2.10 ACTH measurements

The medium of *in vitro* cultured pituitary cells was changed and after 24 h, a conditioned medium sample was collected. ACTH was measured using the Immulite 1000 system (Siemens) according to the manufacturer's instructions. The assay for measuring ACTH was a solid-phase two-step sequential chemiluminescence immunoassay (Siemens Healthineers).

### 2.11 High-fat diet

Ten-week-old male mice (C57BL/6NRj) were caged in a group of 4 animals per cage and allowed to feed ad libitum on a high-fat diet (HFD) or normal diet (ND) (60% kcal from fat or 10% kcal from fat, respectively; D12492 [HFD] and D12450B [ND] Brogaarden, Denmark) for 12 weeks. Their body weight was recorded twice per week. Glucose- and insulin-tolerance tests were conducted in weeks 4 and 5, respectively, as well as in weeks 10 and 11. For glucose- and insulin-tolerance tests, the animals fasted for 5 h and were injected with 1 g/kg of BW glucose solution or 0.75 U/kg of BW insulin (Insuman Infusat, Sanofi) intraperitoneally. After 15, 30, 60, 90, and 120 min, blood glucose (tail vein) was measured with a blood glucose measuring device (Accu-Chek Aviva). After killing the animals using cervical dislocation, adrenal and pituitary glands and hypothalami were excised. The adrenal glands were cleaned from the surrounding fat tissue.

Starting at 6 weeks of age, male mice (C57BL/6 J) were fed a HFD or standard rodent chow for 20 weeks. The HFD (D05031101 M, Research Diets, New Brunswick, NJ, USA) containing 61.3% kcal from fat was used as a DIO model. Weekly weight gain and food intake were monitored. As a standard rodent chow, a V1534 diet (SSNIFF, Soest, Germany) containing 9.0% kcal from fat was used.

### 2.12 Immobilization stress

Stress experiments were as previously described [38]. Briefly, 2-month-old male Nestin-GFP mice were divided into control and experimental (stress) groups (n = 6 per group). The mice from the stress group were placed in individual cages, whereas the control mice were not disturbed. Two days later, at 9 am, the mice were restrained in mouse restrainers (Braintree Scientific) for 2 h. This was repeated over 6 consecutive days. The mice were sacrificed on the last day and their adrenals and hypothalami were collected for RNA isolation.

### 2.13 Immunofluorescence of cryosections

Adrenal glands were fixed (4% PFA, 4 h), cryoprotected (30% sucrose in PBS, 4 °C overnight), embedded in Tissue–Tek Medium (OCT, Sakura Finetek), and stored at −80 °C. Cryosections were sliced to 7 μm thick (Leica CM 1900, Leica Biosystems) and mounted (Superfrost Plus slides, Thermo Fisher Scientific). The slides were immunostained using specific antibodies (Table S1) as previously described. Nuclei were stained with DAPI and the slides were mounted with fluorescent mounting medium (Aqua-Poly/Mount, Polysciences).

### 2.14 Immunofluorescence of paraffin sections

Adult pituitaries were fixed for 16–24 h at room temperature in 10% NBF (Sigma). The samples were dehydrated and stored in 70% ethanol at 4 °C until they embedded. Wax embedding and sectioning were carried out as previously described [49]. Histological sections of 8 μm were used throughout. Slides were deparaffinized in Histoclear (Merck) and rehydrated through a descending graded ethanol series. Antigen retrieval was performed in citrate retrieval buffer pH 6.0 using a Decloaking Chamber NXGEN (Menarini Diagnostics) at 110 °C for 3 min. For paraffin immunofluorescence, sections were blocked in blocking buffer (0.15% glycine, 2 mg/ml of BSA, and 0.1% Triton-X in PBS) with 10% sheep serum (donkey serum for goat SOX2 antibody) for 1 h at room temperature, followed by incubation with primary antibody diluted in blocking buffer with 1% serum at 4 °C overnight. Slides were washed in PBST (0.1% Tween 20 in PBS) and incubated with appropriate biotinylated secondary antibodies or non-biotinylated secondary antibodies in blocking buffer for 1 h at room temperature. Slides were washed in PBST. Slides with biotinylated secondary antibodies were incubated with fluorophore-conjugated Streptavidin (Life Technologies) for 1 h at room temperature together with Hoechst (Life Technologies). After washing in PBST, slides were mounted with VectaMount (Vector Laboratories).

### 2.15 Confocal laser scanning microscopy and fluorescence microscopy

Confocal imaging was performed with a Leica SP5 microscope and LAS X software (Leica). Fluorescence microscopy was conducted with a Zeiss Axiovert 200 M fluorescence microscope, AxioVision software (Zeiss), and a Zeiss Observer Z.1 brightfield microscope with ApoTome and Zen 2012 software. Image processing and analysis were carried out using ImageJ software.

### 2.16 Zonation analysis

To quantify the adrenal gland's different zones, 5–6 sections of whole adrenals were counted per animal (n = 3). The number of cells positive for biotin was related to the adrenocortical cells. The number of cells positive for RGS4 was related to the zG cells. The remaining cells were assumed to be adrenomedullary cells. Cell numbers of different zones in each section were counted and analyzed as the proportion of the total number of all of the cells in each section.

### 2.17 Statistical analysis

Statistical analysis was performed using GraphPad Prism version 5.01 (GraphPad Software Inc.), and statistical significance was determined using one-way or two-way ANOVA followed by a Bonferroni multiple comparison test correction where appropriate. Unpaired two-tailed Student's *t*-test was used when only two means were compared. The significance was defined as: not significant (ns) P > 0.05, ∗P < 0.05, ∗∗P < 0.01, and ∗∗∗P < 0.001.

## 3 Results

### 3.1 *In vitro* differentiation of adrenal progenitors was influenced by insulin

**Figure 1:**
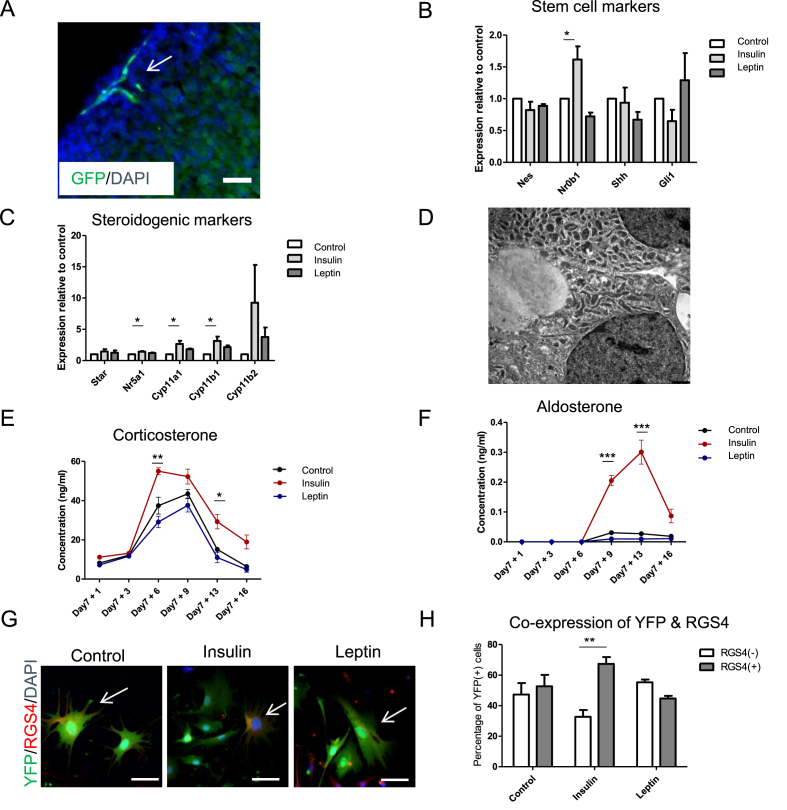
**Insulin affects Nestin(+) adrenocortical progenitors *in vitro*.** (A) Under basal conditions Nestin-GFP(+) cortical cells are mostly located in the adrenal capsule. Scale bar, 100 μm. (B) qRT-PCR showing the relative expression of various stem cell/progenitor markers on day 6 of proliferation and (C) steroidogenic markers on day 6 of differentiation in *in vitro* cultures of adrenocortical progenitors. *P* = 0.0410, 0.0152, 0.0236, and 0.0364 for *Nr0b1*, *Nr5a1*, *Cyp11a1*, and *Cyp11b1* expression for insulin vs control. Data were analyzed by unpaired two-sided *t*-tests. (D) EM image showing a control cell on day 7 of differentiation. (E) Corticosterone and (F) aldosterone levels in the media measured by LC-MS/MS. Data were analyzed by two-way ANOVA and Bonferroni's post-test. (G and H) Tracing of Nestin (+) cells isolated from *Nes-CreERT*^*+/-*^*;Rosa26-eYFP*^*+/+*^ mice, in which recombination was induced *in vitro*. After differentiation for 7 days, cells were immunostained for RGS4 marking zG cells. Double-positive cells are indicated with arrows. *P* = 0.0054 for insulin for RGS4 (+) vs RGS4 (−). Data were analyzed by unpaired two-sided *t*-tests. Representative images are shown. Scale bars, 100 μm. Data in B, C, E, F, and H are presented as mean ± SEM (n ≥ 3, biological replicates). ∗*P* < 0.05, ∗∗*P* < 0.01, and ∗∗∗*P* < 0.001.

Nestin (+) progenitors are present in both the adrenal medulla and cortex. In the adrenal cortex, Nestin (+) progenitors with long processes are mainly located in the capsule or subcapsular region of the adrenal gland (Figure 1A) [38]. We recently showed that Nestin (+) progenitor cells isolated from the adrenal cortex can be cultured and differentiated *in vitro*. Furthermore, descendants of these progenitors are influenced by exposure to Angiotensin II and ACTH [38]. Thus, we isolated adrenocortical cells from Nestin-GFP mice to investigate their reactivity to metabolic challenges. We cultured the cells *in vitro* with or without insulin or leptin. Cultures formed spheroids and were treated for 7 days under proliferative non-adherent conditions (Fig. S2A). Leptin- and insulin-treated spheroids did not show any changes in the expression of *Nes* (Nestin) compared to control cultures (Figure 1B). However, the expression of *Nr0b1* (gene encoding DAX1) increased in the insulin-treated culture, whereas the expression of the stem cell marker *Gli1* and progenitor marker *Shh* was unchanged (Figure 1B).

### 3.2 Steroidogenesis was enhanced by insulin

We next sought to investigate whether insulin or leptin treatment affects adrenocortical differentiation. After 7 days under proliferative conditions with treatment, culture conditions were changed to induce differentiation (Fig. S2B). Treatment with leptin or insulin continued during differentiation. Differentiated adrenocortical cells contained numerous lipid droplets and mitochondria typical of steroid-producing cells of the zona glomerulosa (zG) as observed by electron microscopy (Figure 1D). Co-stimulation of the cells with insulin during 7 days of differentiation led to increased expression of 11β-hydroxylase (*Cyp11b1*), *Cyp11a1*, and *Nr5a1* (gene encoding SF1). Leptin treatment had no effect on the expression of differentiation markers compared to the control (Figure 1C).

Samples of the media from the differentiation cultures were collected over 16 days and steroids were measured. After 6 days of differentiation, the amounts of 11-desoxycorticosterone and corticosterone significantly increased in cultures treated with insulin compared to control cells (Figure 1E). On day 9, the amount of aldosterone was significantly increased in the cultures treated with insulin compared to the control, suggesting enhanced differentiation into zG cells (Figure 1F). Aldosterone levels remained high until day 13 before the concentration decreased. Leptin-stimulated cultures did not differ significantly from controls.

We next investigated whether Nestin (+) cells treated with insulin preferentially differentiate into cells in the zG or zona fasciculata (zF). We isolated adrenocortical cells from *Nes-CreERT;Rosa26-eYFP* mice and treated them with 4OH-tamoxifen over 3 days to induce recombination in Nestin-expressing cells. Descendants of recombined Nestin (+) cells maintained the expression of eYFP irrespective of Nestin expression, allowing lineage tracing of Nestin (+) cells. After further 7 days in proliferative conditions with treatment, the culture conditions were changed to induce differentiation. After 7 days in differentiation conditions, cells were fixed and immunostained for the zG marker RGS4 [50]. The percentage of cells double positive for YFP and RGS4 was significantly increased in the insulin-treated culture. In leptin-treated cultures, we did not observe any significant changes (Figure 1G,H and Fig. S3). The increase in YFP and RGS4 double-positive cells was recorded on day 7 of differentiation. However, 6 days later, a massive increase in aldosterone production occurred. This might have resulted from a simultaneous exponential growth of YFP(+)/RGS4 (+) cells and further amplified aldosterone production.

These results indicated that under basal conditions, adrenocortical Nestin (+) cells were primarily non-committed and slowly differentiated into aldosterone- (zG) and corticosterone-producing (zF) cells. Conversely, high levels of insulin led to enhanced differentiation, particularly into zG cells, accompanied by higher steroid secretion. Not all the RGS4 (+) cells were positive for YFP. Therefore, it must be considered that absolute recombination was not achieved or that other stem/progenitor cells were also able to differentiate into RGS4 (+) cells.

### 3.3 Differentiation of pituitary stem cells was enhanced by insulin

**Figure 2:**
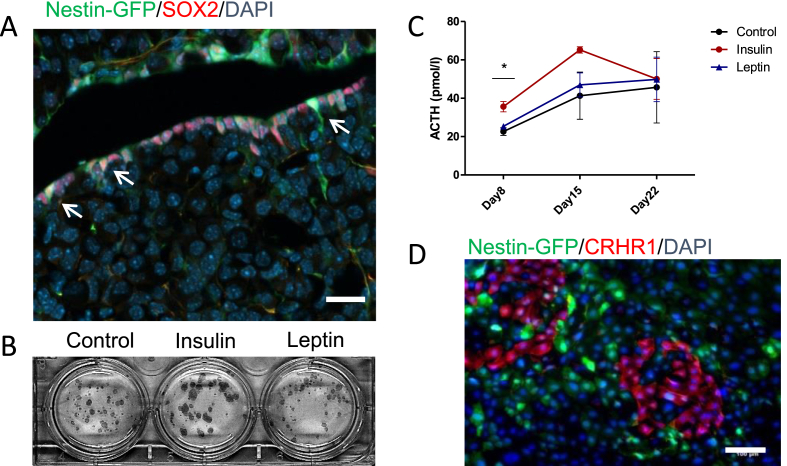
**Insulin affects Nestin(+) progenitors from the anterior pituitary *in vitro*.** (A) Nestin-GFP(+) cells in the anterior lobe of the pituitary gland. Arrows indicate cells double positive for Nestin-GFP and SOX2 in the marginal cell layer. Scale bar, 100 μm. (B) Pituitary Nestin-GFP(+) cells were isolated from the anterior lobe, FACS sorted (1,500 cells/well), and cultured in stem cell supporting media for 30 days. *P* = 0.0494 for day 8 for insulin vs control. Data were analyzed by unpaired two-sided *t*-tests. (C) ACTH content in the media of FACS-sorted Nestin-GFP(+) anterior pituitary cells. (D) CRHR1 immunostaining of FACS-sorted Nestin-GFP(+) anterior pituitary cells 7 days after plating in colony-forming conditions. Scale bar, 100 μm. Data in C are presented as mean ± SEM (n = 3, biological replicates). ∗*P* < 0.05.

As metabolic stress is known to influence the entire HPA axis, we also characterized pituitary progenitors in the presence of leptin or insulin. Bona fide stem cells from the post-natal pituitary gland express SOX2 [33], and a proportion of these co-express Nestin (Figure 2A) [51]. Data on the role of Nestin (+) cells in the anterior pituitary are inconclusive. We isolated these cells from Nestin-GFP animals by fluorescence-activated cell sorting (FACS) and cultured them *in vitro* under clonogenic stem/progenitor-promoting conditions, which only allow uncommitted cells to survive [52]. The cells were cultured in the presence of insulin (4 μg/ml = ∼690 nM) or leptin (1 μg/ml) for 7 days. In the presence of insulin, pituitary progenitors formed larger cell colonies than control cultures, suggesting increased proliferation of these cells (Figure 2B and Figs. S4A–C). No difference in the number of colonies generated was observed between the cultures. To test concentrations of insulin and leptin closer to physiological levels, we isolated cells from the anterior pituitary of WT mice and cultured them *in vitro* in medium containing different concentrations of insulin and leptin. We did not observe any significant differences in the number of colonies (Fig. S4D). A tendency toward larger colonies at the highest concentrations of insulin (100 nM) and leptin (100 ng/ml) was observed, but was not statistically significant (Fig. S4E).

Since ACTH is the anterior pituitary hormone with the highest impact on HPA function, we tested for the presence of ACTH in the media. Significantly higher ACTH levels were measured in the insulin-treated cultures on day 8 (Figure 2C). In the cultures, we examined the expression of the CRH receptor, CRHR1, which is mainly expressed by ACTH-producing cells and a subset of lactotropic cells. We found that CRHR1 (+) cells did not overlap with Nestin-GFP(+) cells, indicating that pituitary Nestin (+) cells were uncommitted (Figure 2D and Fig. S5A). These results indicated that high levels of insulin may lead to an enhanced differentiation into particular ACTH-producing cells and/or that ACTH production accelerates in descendants of pituitary Nestin (+) cells.

### 3.4 The endocrine stress axis changed in obese diabetic (*ob/ob*) mice

Since we observed insulin's influence on the growth and differentiation potential of stem/progenitor cells in the pituitary and adrenal glands *in vitro*, we characterized HPA progenitors in an *in vivo* model of metabolic stress. To elucidate adaptive mechanisms of HPA axis components in various developmental stages and degrees of metabolic syndrome, we analyzed both young and old leptin-deficient *ob/ob* mice in a progressive obesity model. Taking into account that hyperactivity of the HPA axis appears to be most pronounced in female *ob/ob* mice [53], we studied the influence of an early induction of metabolic stress in female mice. We monitored female *ob/ob* mice for 8 weeks. Ten-month old *ob/ob* males were used to study older mice in a more severe chronic stress protocol. In both models, the weight of the obese mice doubled relative to controls (Fig. S7A).

**Figure 3:**
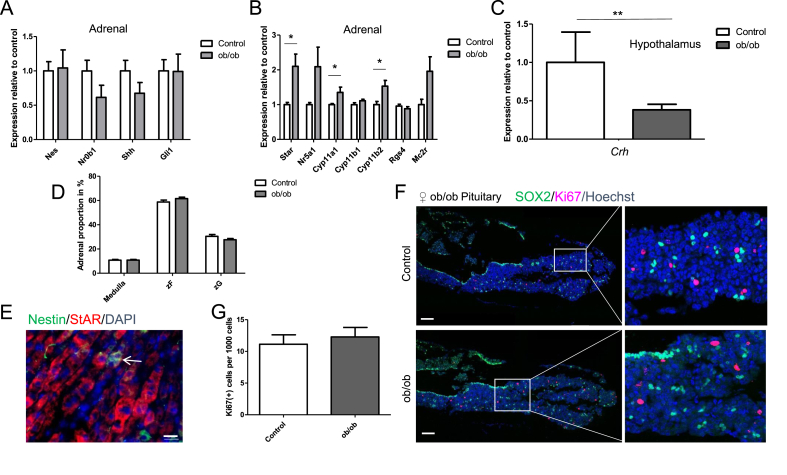
**Leptin-deficiency induces changes in the endocrine stress axis in 8-week-old female *ob/ob* mice.** (A) qRT-PCR analysis showing the relative expression of various stem cell markers and (B) steroidogenic markers plus ACTH receptor in the adrenal gland. *P* = 0.0206, 0.0467, and 0.0267 for *Star*, *Cyp11a1*, *and Cyp11b2* expression for *ob/ob* vs control. Data were analyzed by unpaired two-sided *t*-tests. (C) qRT-PCR analysis showing the relative expression of *Crh* in the hypothalamus. *P* = 0.0021 for *Crh* expression for *ob/ob* vs control. Data were analyzed by unpaired two-sided *t*-tests. (D) Zonation analysis showing the adrenal proportion of zG, zF, and adrenal medulla. (E) Nestin (+) cells migrate and differentiate into StAR (+) cells. Arrow indicates cell double positive for Nestin and StAR. (Scale bar, 20 μm). (F) Immunostaining of SOX2 and Ki67 in the anterior pituitary and (G) quantification of Ki67(+) cells. Scale bars, 100 μm. Representative images are shown. Data in A-D and G are presented as mean ± SEM (n = 6, biological replicates). ∗*P* < 0.05 and ∗∗*P* < 0.01.

In the adrenals of 8-week-old female *ob/ob* mice, gene expression of the stem cell/progenitor markers *Nes*, *Nr0b1*, *Shh*, and *Gli1* was unchanged compared to heterozygous *ob/+* control mice (Figure 3A). These observations were similar to results obtained in a 12-week pre-diabetic HFD mouse model (Fig. S6A and D–E), where the expression of stem/progenitor cell markers was unchanged in the adrenal cortex (Fig. S6B). In the *ob/ob* mice, the expression of steroidogenic markers *Star*, *Cyp11a1*, and *Cyp11b2* significantly increased, whereas the mRNA levels of *Nr5a1*, *Cyp11b1*, *Rgs4*, and *Mc2r* were not significantly altered (Fig. 3B). Similar to the HFD mouse model, the expression of *Crh* in the hypothalamus significantly decreased (Figure 3C and Fig. S6C). These results indicated that leptin deficiency can lead to a higher expression of steroidogenic genes and enzymes in the adrenal gland relative to controls.

### 3.5 Adrenal gland morphology was retained in young female *ob/ob* mice

Studies revealed that adrenal gland weight increases in metabolic syndrome models [54]. To examine if an enlargement of the adrenal results in an increase in the relative size of the zG, zF, or adrenal medulla, we measured the proportion of each zone represented in the adrenal gland. To mark the zG cells, we carried out immunofluorescence staining for RGS4 and used biotin as a marker for the total cortex [55]. Each zone was measured in relation to the total amount of adrenal cells, showing no significant difference in the relative size between *ob/ob* mice and controls (Figure 3D and Fig. S7B).

### 3.6 Adrenocortical Nestin(+) cells differentiated into steroidogenic cells in *ob/ob* mice

We previously showed that under exposure to restraint stress, capsular Nestin (+) cells migrate centripetally from the adrenal capsule toward the adreno-medullary boundary, becoming positive for the steroidogenic markers StAR, CYP11A1, and CYP11B2 [38]. To determine if our chronic metabolic stress protocol also induced differentiation in Nestin (+) cells, we performed immunofluorescence on adrenal sections of young female obese mice. The detection of the migration of Nestin (+) cells was limited due to the narrow time frame of the actual differentiation process and because the expression of Nestin was lost during differentiation [40]. However, *in vitro* we noticed that after differentiation, some StAR (+) cells remained positive for Nestin (Fig. S5B). Similarly, we noticed a few StAR (+) cells with a remaining expression of Nestin in the *ob/ob* mice (Figure 3E and Fig. S7C), indicating that under metabolic stress, Nestin (+) cells mobilized to migrate centripetally and differentiate into steroidogenic cells as observed in restraint stress.

### 3.7 Proliferation was not induced in pituitaries of 8-week-old female and 10-month-old male *ob/ob* mice

**Figure 4:**
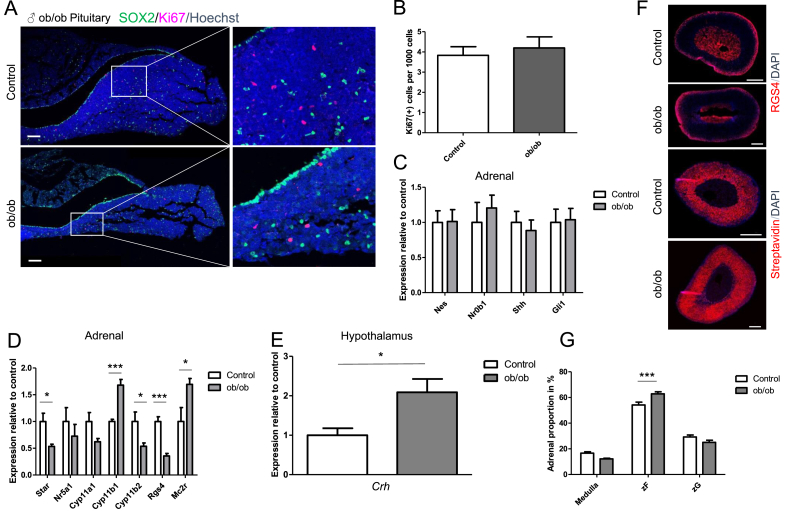
**Diabetic 10-month-old *ob/ob* males show functional and morphological changes in the endocrine stress axis.** (A) Immunostaining of SOX2 and Ki67 in the anterior pituitary and (B) quantification of Ki67(+) cells. Scale bars, 100 μm. Representative images are shown. (C) qRT-PCR analysis showing the relative expression of various stem cell/progenitor markers and (D) steroidogenic markers plus ACTH receptor in the adrenal gland. *P* = 0.0257, 0.0001, 0.0496, 0.0002, and 0.0487 for *Star*, *Cyp11b1*, *Cyp11b2*, *Rgs4*, and *Mc2r* expression for *ob/ob* vs control. Data were analyzed by unpaired two-sided *t*-tests. (E) qRT-PCR analysis of the relative expression of *Crh* in the hypothalamus. *P* = 0.0251 for *Crh* for *ob/ob* vs control. Data were analyzed by unpaired two-sided *t*-tests. (F) Immunofluorescence staining for biotin and RGS4. Background staining appears in the adrenal medulla of RGS4 staining. Scale bars, 200 μm. Representative images are shown. (G) Zonation analysis showing the adrenal proportion of zG, zF, and adrenal medulla. Data were analyzed by two-way ANOVA and Bonferroni's post-test. Data in B-E and G are presented as mean ± SEM (n = 6, biological replicates). ∗*P* < 0.05 and ∗∗∗*P* < 0.001.

To assess whether the number of cycling cells increased in the pituitary of obese *ob/ob* mice, we carried out immunofluorescence staining with antibodies against Ki67. We did not observe any difference in the percentage of Ki67(+) cells. Nestin (+) pituitary progenitors might represent a subpopulation of SOX2 (+) stem cells, which also reside in the anterior lobe and play an important role in long-term physiological maintenance of the adult pituitary gland [33]. Hence, to assess the number of cycling cells among the stem/progenitor compartment, we performed double immunofluorescence staining for SOX2 and Ki67. Only a minor number of SOX2 (+) cells were double positive for Ki67 (Figure 3F,G). Similarly, we did not observe any differences in cycling cells in older *ob/ob* animals. Moreover, the appearance and localization of SOX2 (+) cells were unchanged (Figure 4A,B).

### 3.8 Severe obesity in 10-month-old *ob/ob* mice was associated with changes in gene expression and morphological aspects in the adrenal gland

In the 10-month-old male *ob/ob* mice, we did not observe any changes in gene expression associated with stem/progenitor cells in the adrenal gland (Figure 4C). However, in contrast to young female *ob/ob* mice, mRNA levels of *Star*, *Cyp11b2*, and *Rgs4* decreased, while the expression of *Cyp11b1* and *Mc2r* increased (Figure 4D). Gene expression of *Nr5a1* and *Cyp11a1* did not change significantly. In the hypothalamus, the amount of *Crh* increased in *ob/ob* mice, contrary to the young *ob/ob* model (Figure 4E). Morphological analyses revealed an enlargement of the adrenal cortex in *ob/ob* mice, which was essentially due to the expansion of the zF (Figure 4F,G).

### 3.9 Genes involved in energy balance were similarly regulated by different chronic stress models

**Figure 5:**
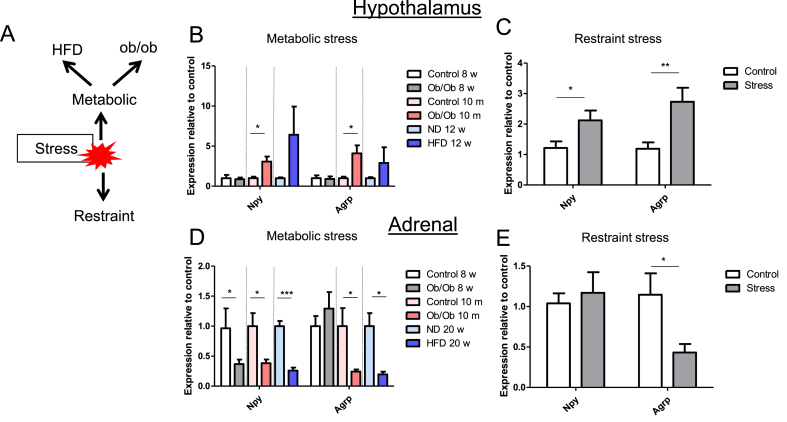
**Chronic stress affects genes involved in energy balance.** (A) Schematic representation of different chronic stress models. (B) qRT-PCR analysis showing the relative expression of hypothalamic *Npy* and *Agrp* in different metabolic stress models and (C) restraint stress. *P* = 0.0182 for *Npy* expression and *P* = 0.0217 for *Agrp* expression for *ob/ob* vs control in metabolic stress. *P* = 0.0285 for *Npy* expression and *P* = 0.0053 for *Agrp* expression for stress vs control in restraint stress. Data were analyzed by unpaired two-sided *t*-tests. (D) qRT-PCR analysis showing the relative expression of *Agrp* and *Npy* in the adrenal glands of different metabolic stress models and (D) restraint stress. *P* = 0.0138, 0.0343, and 0.0008 from left to right for *Npy* expression and *P* = 0.0493 and 0.0331 for *Agrp* expression for “metabolic stress” vs control. *P* = 0.0314 for *Agrp* expression for stress vs control in restraint stress. Data were analyzed by unpaired two-sided *t*-tests. Data in B-E are presented as mean ± SEM (n ≥ 6, biological replicates). ∗P < 0.05 and ∗∗P < 0.01.

We did not observe any alterations in the gene expression of stem/progenitor cell markers in our animal models for metabolic stress. However, as we observed a weight increase and pre-diabetes in both *ob/ob* and HFD models, we explored the expression of genes involved in appetite regulation and determine how their expression compares to other forms of stress, such as a restraint stress model, in which we previously observed increased differentiation of progenitors (Figure 5A) [38].

The appetite-regulating factors AgRP and NPY are not only expressed in the hypothalamus, but also by chromaffin cells in the adrenal medulla [56]. Therefore, we assessed the expression of these genes in the hypothalamus and adrenal gland during metabolic and restraint stress.

In the hypothalamus, we observed similar gene expression patterns of *Agrp* and *Npy* among our different stress models. The expression of *Npy* and *Agrp* was unchanged in young female *ob/ob* mice, whereas the expression of *Npy* and *Agrp* increased in the 10-month-old male *ob/ob* mice (Figure 5B). Both *Npy* and *Agrp* had elevated expression levels after 12 weeks on a HFD, although these did not reach significance (Figure 5B). A significant elevation of *Npy* and *Agrp* was observed in the hypothalamus in a restraint stress model, in which 8-week-old male C57BL/6 N mice were restrained for 2 h per day for six consecutive days (Figure 5C).

In the adrenals of young female *ob/ob* mice, the expression of *Npy* decreased compared to control animals, whereas no significant difference in the expression of *Agrp* was observed (Figure 5D). In the 10-month-old male *ob/ob* mice, the expression of adrenal *Agrp* and *Npy* was lower compared to controls (Figure 5D). In a previously described 20-week HFD model [57], the expression of *Agrp* and *Npy* in whole adrenals also decreased significantly (Figure 5D). Restraint stress led to a significant decrease in the expression of *Agrp*, while the expression of *Npy* was unaltered in the adrenal (Figure 5E).

## 4 Discussion

In the present study, we have shown that *in vitro* stimulation with insulin altered the growth and differentiation behavior of Nestin (+) cells in both the adrenal cortex and anterior lobe of the pituitary gland. We used concentrations of insulin that were very high compared to physiological concentrations [58] as it was previously shown for neural stem cells that to reactivate these from quiescence high local concentrations of insulin or insulin-like growth factors (IGFs) are required [59]. Furthermore, the concentrations we used are those typically used in culture media to grow neurospheres [60]. The same is the case for leptin, where the concentrations in culture media are typically higher than physiological concentrations [61,62]. This also fits with our observations *in vitro*, in which we did not observe any effect on pituitary stem cells at low (physiological) concentrations of insulin or leptin. At physiological concentrations, insulin activates the insulin receptor, leading to the activation of the PI3K/Akt pathway. At higher concentrations, signaling can also act via IGF receptors [58], suggesting that in our study, signaling was via IGF receptors instead of insulin receptors. For example, hippocampal astrocytes produce IGF-1, which promotes neural stem cell (NSC) proliferation in mammalian adult brains [63,64]. IGF-1 is also expressed in neurons and NSCs, and during neurogenesis, its expression in the brain is much higher than in the systemic circulation [59].

**Figure 6:**
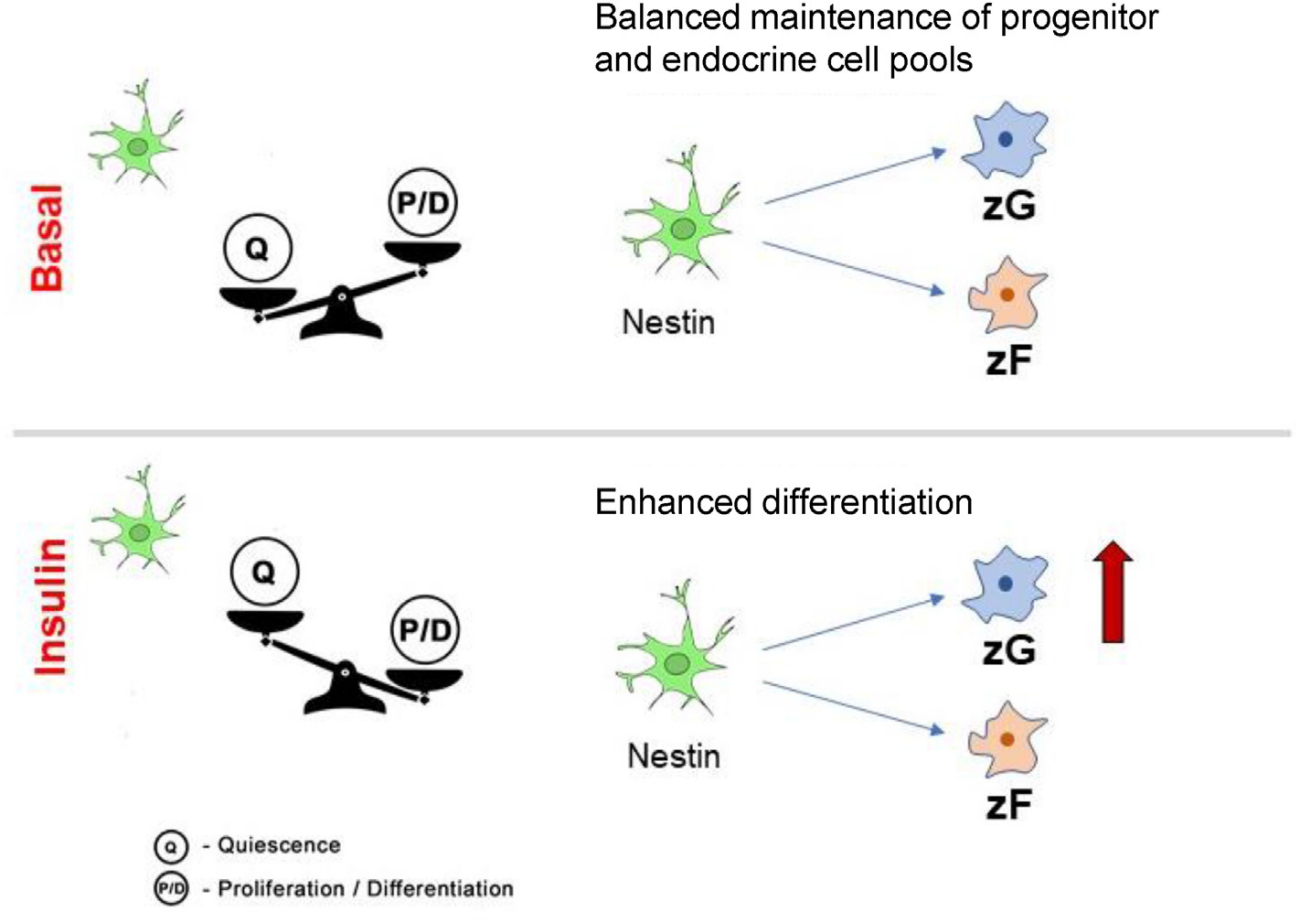
**Proposed model of the function of Nestin(+) progenitors under the influence of insulin.** Under basal conditions, adrenocortical Nestin (+) cells are primarily uncommitted and slowly differentiate into aldosterone- (zG) and corticosterone-producing (zF) cells. In contrast, high levels of insulin lead to an enhanced differentiation into zG cells, which is accompanied by higher steroid secretion.

At the transcriptomic level, we did not observe any changes in the expression of *Nes* in the proliferation cultures. However, lineage tracing revealed that adrenocortical Nestin (+) cells showed an enhanced differentiation into particularly zG cells in the presence of insulin (Figure 6). During the differentiation of adrenocortical progenitors, steroidogenesis augmented as a dramatic increase in adrenal steroids, especially aldosterone, was observed after the addition of insulin. This might suggest a direct link between obesity/T2D and hypertension. Human adipocytes have also been shown to secrete potent mineralocorticoid-releasing factors [65], indicating an additional connection between obesity and hypertension. Moreover, Nestin (+) pituitary cells and their progeny featured an enhanced production of ACTH in the presence of insulin, suggesting that different endocrine progenitor subpopulations are susceptible to metabolic stress. This was supported by the fact that in Nestin (+) NSCs, the differentiation fate was affected by streptozotocin, a compound causing insulin resistance [66]. Furthermore, insulin itself has been shown to regulate proliferation and differentiation in NSCs [60]. Leptin has previously been shown to affect the development of murine embryonic stem cells *in vitro* [67]. However, as in the adrenal NCI–H295 tumor cell line and primary adrenal cells [68], we did not observe any significant effects of leptin on endocrine pituitary or adrenocortical progenitors.

A T2D model that has been widely studied is *ob/ob* mice (leptin deficiency), as their morbid obesity and T2D are similar to those observed in humans, making them a good model without any interfering factors [69]. Although their primary phenotype is obesity, these mice also display insulin resistance and some degree of dyslipidemia, so can be considered a reliable and standardized model of metabolic syndrome [70]. Various studies suggest the concept of sexual- and age-related dimorphism in diabetic/obese mice presenting higher susceptibility to metabolic dysfunction in males [[70], [71], [72], [73]]. However, hyperactivity of the HPA axis appears to be most pronounced in *ob/ob* females [53]. Our observations consistently indicated HPA axis hyperactivation already observed in 8-week-old female *ob/ob* mice, since all of the steroidogenic genes were upregulated in these animals. Conversely, in the severely diseased 10-month-old obese mice, a downregulation of *Star* and *Cyp11b2* was detected. We inconsistently observed an expansion of the adrenal cortex after 10 months of progressive obesity. A possible explanation might be chronic inflammation, which typically accompanies symptoms of obesity, especially in long-lasting manifestations [74,75]. Thereby, lipid accumulation and the release of various factors including cytokines, peptides, and neurotransmitters might be responsible for the downregulation of steroidogenic genes in old mice [23,24].

Both our systems were chronic stress models running over several months. This might explain why we did not observe any significant changes in the gene expression of stem/progenitor cells at the end of the experiments. This was supported by our previous restraint stress studies, in which we observed an extended proliferation of progenitors in short stress experiments, but after a resting period, homeostasis was re-achieved [38,43]. To reveal clear differences at the stem cell level, an acute metabolic stress experiment would be recommended, although not possible in an obesity model. Another possibility would be to use tamoxifen-inducible Cre lineage-tracing mice in obesity models to follow stem and progenitor cells. However, in our experience in the current and previous studies, it was difficult to achieve full recombination both *in vitro* and *in vivo* [38].

In general, stress is defined as a challenge to an individual's homeostasis [34]. The bidirectional interplay between obesity as a form of stress and altered HPA function during the development of obesity makes it difficult to diagnose a cause or consequence. Stress affects the eating behavior of rodents and humans, suggesting that the regulation of energy balance and stress response is a coupled physiological process. Long-lasting satiety signals such as insulin and leptin usually act to decrease AgRP in the hypothalamus. Nevertheless, hypothalamic AgRP can also be upregulated in leptin-deficient mice irrespective of fasting [76], which we also observed in the 10-month-old male *ob/ob* mice. Consistent with our restraint stress model, we ascertained higher *Npy* and *Agrp* expression levels that consequently may lead to increased stress-induced food intake and therefore an increased vulnerability to developing metabolic disorders. AgRP is likely to have an autocrine/paracrine role in the adrenal glands, with adrenal-derived AgRP being regulated by glucocorticoids and blocking the induction of corticosterone secretion by alpha-melanocyte-stimulating hormone [77,78]. Both the 10-month-old male *ob/ob* mice and 20-week HFD model showed lower expression of *Npy* and *Agrp* in adrenal glands. Furthermore, both metabolic and restraint stress led to an attenuated expression of *Agrp* in adrenal glands, suggesting shared underlying adaptive stress response mechanisms. This emphasizes the complex interplay between nutritional status in mammals and stress hormone regulation. Not only internal stressors, such as factors regulating feeding behavior, are capable of shaping the endocrine stress axis, but an inverse effect also exists. This at least in part explains the shared biology of obesity and maladaptive processes in the endocrine stress response [79].

## 5 Conclusions

In conclusion, we have shown that insulin activates stem cells of the HPA axis by enhancing their proliferation and differentiation, leading to augmented hormone production. We have also demonstrated that animal models of chronic metabolic stress lead to hyperactivation of the HPA axis. We recently introduced the concept of stress-inducible stem cells and discussed if stress affects stem/progenitor cells through epigenetic modifications whereby they will be predisposed to adult disease [41]. Ongoing and continuous shaping and transformation of the HPA axis through the induction of subpopulations of progenitors might then explain the influence of early life stress on mental and metabolic illness in adulthood, which was previously reported [[80], [81], [82], [83]].

## Contributions

M.W., H.M., C.L.A., S.R.B., and C.S. designed this study. M.W., I.B., L.D.S., A. Sa., P.S., H.B., and M.P. conducted the research. M.W., I.B., L.D.S., A. Sa., A. Sc., A.J.F.K., S.R.B, and C.S. analyzed the data. M.W. and C.S. wrote the paper. S.R.B. and C.S. supervised the study.

## Acknowledgements

We thank Uta Lehnert and Linda Friedrich for technical assistance. Erwin Weiss from the FACS facility at the CMCB Technology Platform at TU Dresden is thanked for his assistance with the cell sorting.

This work was supported by the 10.13039/501100001659Deutsche Forschungsgemeinschaft (DFG, German Research foundation) project no. 314061271, TRR 205/1: “The Adrenal: Central Relay in Health and Disease” and project no. 288034826, IRTG 2251: “Immunological and Cellular Strategies in Metabolic Disease”.

## Conflict of interest

The authors declare no competing interests.

## Appendix A Supplementary data

Figure S1 **Gating strategy for fluorescent-activated cell sorting of pituitary stem cells.** The anterior pituitary was isolated from Nestin-GFP mice and dissociated into a single-cell suspension. Living cells were identified as PI(−). GFP(+) and GFP(−) single cell populations were sorted (marked with red arrows). Figure S1

Figure S2 ***In vitro* cultures of adrenocortical progenitors.** (A) Adrenocortical cells from Nestin-GFP mice were isolated and cultured under low-attachment conditions in the presence of insulin or leptin as indicated. (B) On day 7, the culture conditions were changed and differentiation was induced. Stimulation with insulin and leptin continued. The expression of GFP was tracked throughout the experiment and representative images are shown. Figure S2

Figure S3 ***In vitro* lineage tracing of adrenocortical progenitors.** Tracing of YFP(+) cells isolated from *Nes-CreERT*^*+/-*^*;Rosa26-eYFP*^*+/+*^ mice, in which recombination was induced *in vitro*. After differentiation for 7 days, the cells were immunostained for RGS4 marking zG cells and YFP marking Nestin-derived cells. Double-positive cells are indicated with arrows. Secondary antibodies alone were observed in Ab control. Representative images are shown. Figure S3

Figure S4 ***In vitro* culture of pituitary progenitors.** (A) Cells from the pituitary anterior lobe of Nestin-GFP mice were isolated and cultured in stem cell supporting medium in the presence of insulin or leptin as indicated. (B) Single colony. (C) Average colony area. Data in C are presented as mean ± SEM (n = 3; biological replicates). Data were analyzed by one-way ANOVA and Bonferroni's post-test. ∗∗∗P < 0.001. (D) Cells from the pituitary anterior lobe of WT mice were isolated and cultured in stem cell supporting medium I containing different concentrations of insulin or leptin as indicated. The number of colonies per well was counted (n = 6; biological replicates). Data were analyzed by unpaired two-sided *t*-tests. (E) Average colony area (n = 3; biological replicates). Data were analyzed by unpaired two-sided *t*-tests. *P* = 0.0116 for leptin (100 ng/ml vs 5 ng/ml). Figure S4

Figure S5 **Antibody validation.** (A) The antibody against CRHR1 was optimized and validated on pituitary stem cell colonies isolated from Nestin-GFP mice and cultured *in vitro*. Secondary antibodies alone were observed in Ab control. (B) Antibodies against StAR and Nestin were optimized and validated on adrenocortical progenitors, which were isolated from WT mice and differentiated into steroidogenic cells *in vitro*. Secondary antibodies alone were observed in Ab control. Figure S5

Figure S6 **HFD experiment.** (A) Weight development of mice on 12 weeks of ND and HFD. Data were analyzed by two-way ANOVA and Bonferroni's post-test. (B) qRT-PCR analysis showing the relative expression of various stem cell/progenitor markers. (C) qRT-PCR analysis of the relative expression of *Crh* in the hypothalamus. (D) Glucose-tolerance tests were performed after 4 and 10 weeks. Data were analyzed by two-way ANOVA and Bonferroni's post-test. (E) Insulin-tolerance tests were performed after 5 and 11 weeks. *P* = 0.0480 and 0.0060 for 60 and 120 min for HFD vs ND for 5 weeks. *P* = 0.0431 for 30 min for HFD vs ND for 11 weeks. Data were analyzed by unpaired two-sided *t*-tests. Data are presented as mean ± SEM (n ≥ 6, biological replicates). ∗P < 0.05, ∗∗P < 0.01, and ∗∗∗P < 0.001. Figure S6

Figure S7 **Eight-week-old *ob/ob* females and 10-month-old *ob/ob* males.** (A) Final body weight of obese *ob/ob* mice and their controls. Data were analyzed by unpaired two-sided *t*-tests. (B) Representative images of immunostaining for zonation analysis of 8-week-old female *ob/ob* mice are shown. Scale bars, 200 μm. (C) Nestin(+) cells migrated and differentiated into StAR(+) cells. Arrow indicates double-positive cell for Nestin and StAR. Scale bar, 20 μm. Representative images are shown. ∗∗∗P < 0.001. Figure S7

## References

[1] Hackett R.A., Steptoe A. (2017). Type 2 diabetes mellitus and psychological stress - a modifiable risk factor. Nature Reviews Endocrinology.

[2] Sominsky L., Spencer S.J. (2014). Eating behavior and stress: a pathway to obesity. Front Psychol.

[3] Afrisham R., Paknejad M., Soliemanifar O., Sadegh-Nejadi S., Meshkani R., Ashtary-Larky D. (2019). The influence of psychological stress on the initiation and progression of diabetes and cancer. International Journal of Endocrinology and Metabolism.

[4] Joseph J.J., Golden S.H. (2017). Cortisol dysregulation: the bidirectional link between stress, depression, and type 2 diabetes mellitus. Annals of the New York Academy of Sciences.

[5] Rosmond R. (2005). Role of stress in the pathogenesis of the metabolic syndrome. Psychoneuroendocrinology.

[6] Bornstein S.R., Uhlmann K., Haidan A., Ehrhart-Bornstein M., Scherbaum W.A. (1997). Evidence for a novel peripheral action of leptin as a metabolic signal to the adrenal gland: leptin inhibits cortisol release directly. Diabetes.

[7] Kinyua A.W., Doan K.V., Yang D.J., Huynh M.K.Q., Choi Y.H., Shin D.M. (2018). Insulin regulates adrenal steroidogenesis by stabilizing SF-1 activity. Scientific Reports.

[8] Pralong F.P., Roduit R., Waeber G., Castillo E., Mosimann F., Thorens B. (1998). Leptin inhibits directly glucocorticoid secretion by normal human and rat adrenal gland. Endocrinology.

[9] Berger I., Werdermann M., Bornstein S.R., Steenblock C. (2019). The adrenal gland in stress - adaptation on a cellular level. The Journal of Steroid Biochemistry and Molecular Biology.

[10] Currie P.J. (2003). Integration of hypothalamic feeding and metabolic signals: focus on neuropeptide. Y. Appetite..

[11] Heinrichs S.C., Menzaghi F., Pich E.M., Hauger R.L., Koob G.F. (1993). Corticotropin-releasing factor in the paraventricular nucleus modulates feeding induced by neuropeptide Y. Brain Research.

[12] Heinrichs S.C., Richard D. (1999). The role of corticotropin-releasing factor and urocortin in the modulation of ingestive behavior. Neuropeptides.

[13] Richard D., Lin Q., Timofeeva E. (2002). The corticotropin-releasing factor family of peptides and CRF receptors: their roles in the regulation of energy balance. European Journal of Pharmacology.

[14] Ehrhart-Bornstein M., Arakelyan K., Krug A.W., Scherbaum W.A., Bornstein S.R. (2004). Fat cells may be the obesity-hypertension link: human adipogenic factors stimulate aldosterone secretion from adrenocortical cells. Endocrine Research.

[15] Konno J., Yoshida S., Ina A., Ohmomo H., Shutoh F., Nogami H. (2008). Upregulated expression of neuropeptide Y in hypothalamic-pituitary system of rats by chronic dexamethasone administration. Neurosci Res.

[16] Savontaus E., Conwell I.M., Wardlaw S.L. (2002). Effects of adrenalectomy on AGRP, POMC, NPY and CART gene expression in the basal hypothalamus of fed and fasted rats. Brain Research.

[17] Shimizu H., Arima H., Watanabe M., Goto M., Banno R., Sato I. (2008). Glucocorticoids increase neuropeptide Y and agouti-related peptide gene expression via adenosine monophosphate-activated protein kinase signaling in the arcuate nucleus of rats. Endocrinology.

[18] Bergmann N., Gyntelberg F., Faber J. (2014). The appraisal of chronic stress and the development of the metabolic syndrome: a systematic review of prospective cohort studies. Endocr Connect.

[19] Tamashiro K.L., Sakai R.R., Shively C.A., Karatsoreos I.N., Reagan L.P. (2011). Chronic stress, metabolism, and metabolic syndrome. Stress: The International Journal on the Biology of Stress.

[20] Yam K.Y., Ruigrok S.R., Ziko I., De Luca S.N., Lucassen P.J., Spencer S.J. (2017). Ghrelin and hypothalamic NPY/AgRP expression in mice are affected by chronic early-life stress exposure in a sex-specific manner. Psychoneuroendocrinology.

[21] Jequier E. (2002). Leptin signaling, adiposity, and energy balance. Annals of the New York Academy of Sciences.

[22] Zakrzewska K.E., Cusin I., Sainsbury A., Rohner-Jeanrenaud F., Jeanrenaud B. (1997). Glucocorticoids as counterregulatory hormones of leptin: toward an understanding of leptin resistance. Diabetes.

[23] Bornstein S.R., Rutkowski H., Vrezas I. (2004). Cytokines and steroidogenesis. Molecular and Cellular Endocrinology.

[24] Ehrhart-Bornstein M., Hinson J.P., Bornstein S.R., Scherbaum W.A., Vinson G.P. (1998). Intraadrenal interactions in the regulation of adrenocortical steroidogenesis. Endocrine Reviews.

[25] Bestetti G.E., Abramo F., Guillaume-Gentil C., Rohner-Jeanrenaud F., Jeanrenaud B., Rossi G.L. (1990). Changes in the hypothalamo-pituitary-adrenal axis of genetically obese fa/fa rats: a structural, immunocytochemical, and morphometrical study. Endocrinology.

[26] Wolkersdorfer G.W., Bornstein S.R. (1998). Tissue remodelling in the adrenal gland. Biochemical Pharmacology.

[27] Burgio E., Lopomo A., Migliore L. (2015). Obesity and diabetes: from genetics to epigenetics. Molecular Biology Reports.

[28] Castellano-Castillo D., Ramos-Molina B., Cardona F., Queipo-Ortuno M.I. (2020). Epigenetic regulation of white adipose tissue in the onset of obesity and metabolic diseases. Obesity Reviews.

[29] Crujeiras A.B., Campion J., Diaz-Lagares A., Milagro F.I., Goyenechea E., Abete I. (2013). Association of weight regain with specific methylation levels in the NPY and POMC promoters in leukocytes of obese men: a translational study. Regulatory Peptides.

[30] Lesseur C., Armstrong D.A., Paquette A.G., Koestler D.C., Padbury J.F., Marsit C.J. (2013). Tissue-specific Leptin promoter DNA methylation is associated with maternal and infant perinatal factors. Molecular and Cellular Endocrinology.

[31] Janesick A., Blumberg B. (2012). Obesogens, stem cells and the developmental programming of obesity. Int J Androl.

[32] Sookoian S., Gianotti T.F., Burgueno A.L., Pirola C.J. (2013). Fetal metabolic programming and epigenetic modifications: a systems biology approach. Pediatric Research.

[33] Andoniadou C.L., Matsushima D., Mousavy Gharavy S.N., Signore M., Mackintosh A.I., Schaeffer M. (2013). Sox2(+) stem/progenitor cells in the adult mouse pituitary support organ homeostasis and have tumor-inducing potential. Cell Stem Cell.

[34] Bornstein S.R., Berger I., Scriba L., Santambrogio A., Steenblock C. (2019). Adrenal cortex–medulla interactions in adaptation to stress and disease. Curr Opin Endocrin Metabol Res.

[35] Finco I., Lerario A.M., Hammer G.D. (2018). Sonic hedgehog and WNT signaling promote adrenal gland regeneration in male mice. Endocrinology.

[36] Youssef M., Atsak P., Cardenas J., Kosmidis S., Leonardo E.D., Dranovsky A. (2019). Early life stress delays hippocampal development and diminishes the adult stem cell pool in mice. Scientific Reports.

[37] Steenblock C., Rubin de Celis M.F., Androutsellis-Theotokis A., Sue M., Delgadillo Silva L.F., Eisenhofer G. (2017). Adrenal cortical and chromaffin stem cells: is there a common progeny related to stress adaptation?. Molecular and Cellular Endocrinology.

[38] Steenblock C., Rubin de Celis M.F., Delgadillo Silva L.F., Pawolski V., Brennand A., Werdermann M. (2018). Isolation and characterization of adrenocortical progenitors involved in the adaptation to stress. Proceedings of the National Academy of Sciences of the U S A.

[39] Chetty S., Friedman A.R., Taravosh-Lahn K., Kirby E.D., Mirescu C., Guo F. (2014). Stress and glucocorticoids promote oligodendrogenesis in the adult hippocampus. Molecular Psychiatry.

[40] Bornstein S.R., Berger I., Steenblock C. (2020). Are Nestin-positive cells responsive to stress?. Stress: The International Journal on the Biology of Stress.

[41] Bornstein S.R., Steenblock C., Chrousos G.P., Schally A.V., Beuschlein F., Kline G. (2019). Stress-inducible-stem cells: a new view on endocrine, metabolic and mental disease?. Molecular Psychiatry.

[42] Mignone J.L., Kukekov V., Chiang A.S., Steindler D., Enikolopov G. (2004). Neural stem and progenitor cells in nestin-GFP transgenic mice. The Journal of Comparative Neurology.

[43] Rubin de Celis M.F., Garcia-Martin R., Wittig D., Valencia G.D., Enikolopov G., Funk R.H. (2015). Multipotent glia-like stem cells mediate stress adaptation. Stem Cells.

[44] Burns K.A., Ayoub A.E., Breunig J.J., Adhami F., Weng W.L., Colbert M.C. (2007). Nestin-CreER mice reveal DNA synthesis by nonapoptotic neurons following cerebral ischemia hypoxia. Cerebral Cortex.

[45] Srinivas S., Watanabe T., Lin C.S., William C.M., Tanabe Y., Jessell T.M. (2001). Cre reporter strains produced by targeted insertion of EYFP and ECFP into the ROSA26 locus. BMC Developmental Biology.

[46] Livak K.J., Schmittgen T.D. (2001). Analysis of relative gene expression data using real-time quantitative PCR and the 2(-Delta Delta C(T)) Method. Methods.

[47] Mangelis A., Dieterich P., Peitzsch M., Richter S., Juhlen R., Hubner A. (2016). Computational analysis of liquid chromatography-tandem mass spectrometric steroid profiling in NCI H295R cells following angiotensin II, forskolin and abiraterone treatment. The Journal of Steroid Biochemistry and Molecular Biology.

[48] Peitzsch M., Dekkers T., Haase M., Sweep F.C., Quack I., Antoch G. (2015). An LC-MS/MS method for steroid profiling during adrenal venous sampling for investigation of primary aldosteronism. The Journal of Steroid Biochemistry and Molecular Biology.

[49] Gaston-Massuet C., Andoniadou C.L., Signore M., Jayakody S.A., Charolidi N., Kyeyune R. (2011). Increased Wingless (Wnt) signaling in pituitary progenitor/stem cells gives rise to pituitary tumors in mice and humans. Proceedings of the National Academy of Sciences of the U S A.

[50] Romero D.G., Zhou M.Y., Yanes L.L., Plonczynski M.W., Washington T.R., Gomez-Sanchez C.E. (2007). Regulators of G-protein signaling 4 in adrenal gland: localization, regulation, and role in aldosterone secretion. Journal of Endocrinology.

[51] Gleiberman A.S., Michurina T., Encinas J.M., Roig J.L., Krasnov P., Balordi F. (2008). Genetic approaches identify adult pituitary stem cells. Proceedings of the National Academy of Sciences of the U S A.

[52] Andoniadou C.L., Gaston-Massuet C., Reddy R., Schneider R.P., Blasco M.A., Le Tissier P. (2012). Identification of novel pathways involved in the pathogenesis of human adamantinomatous craniopharyngioma. Acta Neuropathologica.

[53] McGinnis R., Walker J., Margules D., Aird F., Redei E. (1992). Dysregulation of the hypothalamus-pituitary-adrenal Axis in male and female, genetically obese (ob/ob) mice. Journal of Neuroendocrinology.

[54] Topal F., Goren H., Yucel F., Sahinturk V., Aydar Y. (2019). Effect of consuming high-fat diet on the morphological parameters of adrenal gland. Bratislavske Lekarske Listy.

[55] Paul A., Laufer E. (2011). Endogenous biotin as a marker of adrenocortical cells with steroidogenic potential. Molecular and Cellular Endocrinology.

[56] Gupta R., Ma Y., Wang M., Whim M.D. (2017). AgRP-expressing adrenal chromaffin cells are involved in the sympathetic response to fasting. Endocrinology.

[57] Langbein H., Hofmann A., Brunssen C., Goettsch W., Morawietz H. (2015). Impact of high-fat diet and voluntary running on body weight and endothelial function in LDL receptor knockout mice. Atherosclerosis Supplements.

[58] Ziegler A.N., Levison S.W., Wood T.L. (2015). Insulin and IGF receptor signalling in neural-stem-cell homeostasis. Nature Reviews Endocrinology.

[59] Ding W.Y., Huang J., Wang H. (2020). Waking up quiescent neural stem cells: molecular mechanisms and implications in neurodevelopmental disorders. PLoS Genetics.

[60] Chirivella L., Kirstein M., Ferron S.R., Domingo-Muelas A., Durupt F.C., Acosta-Umanzor C. (2017). Cyclin-dependent kinase 4 regulates adult neural stem cell proliferation and differentiation in response to insulin. Stem Cells.

[61] Gajewski M., Gajewska J., Rzodkiewicz P., Wojtecka-Lukasik E. (2016). Influence of exogenous leptin on redox homeostasis in neutrophils and lymphocytes cultured in synovial fluid isolated from patients with rheumatoid arthritis. Reumatologia.

[62] Kamp V.M., Langereis J.D., van Aalst C.W., van der Linden J.A., Ulfman L.H., Koenderman L. (2013). Physiological concentrations of leptin do not affect human neutrophils. PloS One.

[63] Anderson M.F., Aberg M.A., Nilsson M., Eriksson P.S. (2002). Insulin-like growth factor-I and neurogenesis in the adult mammalian brain. Brain Res Dev Brain Res.

[64] Song H., Stevens C.F., Gage F.H. (2002). Astroglia induce neurogenesis from adult neural stem cells. Nature.

[65] Ehrhart-Bornstein M., Lamounier-Zepter V., Schraven A., Langenbach J., Willenberg H.S., Barthel A. (2003). Human adipocytes secrete mineralocorticoid-releasing factors. Proceedings of the National Academy of Sciences of the U S A.

[66] Sun P., Ortega G., Tan Y., Hua Q., Riederer P.F., Deckert J. (2018). Streptozotocin impairs proliferation and differentiation of adult hippocampal neural stem cells in vitro-correlation with alterations in the expression of proteins associated with the insulin system. Frontiers in Aging Neuroscience.

[67] Taskin A.C., Kocabay A., Ebrahimi A., Karahuseyinoglu S., Sahin G.N., Ozcimen B. (2019). Leptin treatment of in vitro cultured embryos increases outgrowth rate of inner cell mass during embryonic stem cell derivation. In Vitro Cellular & Developmental Biology Animal.

[68] Glasow A., Bornstein S.R. (2000). Leptin and the adrenal gland. European Journal of Clinical Investigation.

[69] Coleman D.L. (1978). Obese and diabetes: two mutant genes causing diabetes-obesity syndromes in mice. Diabetologia.

[70] Kennedy A.J., Ellacott K.L., King V.L., Hasty A.H. (2010). Mouse models of the metabolic syndrome. Dis Model Mech.

[71] Hwang L.L., Wang C.H., Li T.L., Chang S.D., Lin L.C., Chen C.P. (2010). Sex differences in high-fat diet-induced obesity, metabolic alterations and learning, and synaptic plasticity deficits in mice. Obesity.

[72] Nishikawa S., Yasoshima A., Doi K., Nakayama H., Uetsuka K. (2007). Involvement of sex, strain and age factors in high fat diet-induced obesity in C57BL/6J and BALB/cA mice. Experimental Animals.

[73] Stubbins R.E., Holcomb V.B., Hong J., Nunez N.P. (2012). Estrogen modulates abdominal adiposity and protects female mice from obesity and impaired glucose tolerance. European Journal of Nutrition.

[74] Ellulu M.S., Patimah I., Khaza'ai H., Rahmat A., Abed Y. (2017). Obesity and inflammation: the linking mechanism and the complications. Archives of Medical Science.

[75] Stepien M., Stepien A., Wlazel R.N., Paradowski M., Banach M., Rysz J. (2014). Obesity indices and inflammatory markers in obese non-diabetic normo- and hypertensive patients: a comparative pilot study. Lipids in Health and Disease.

[76] Shutter J.R., Graham M., Kinsey A.C., Scully S., Luthy R., Stark K.L. (1997). Hypothalamic expression of ART, a novel gene related to agouti, is up-regulated in obese and diabetic mutant mice. Genes & Development.

[77] Dhillo W.S., Small C.J., Gardiner J.V., Bewick G.A., Whitworth E.J., Jethwa P.H. (2003). Agouti-related protein has an inhibitory paracrine role in the rat adrenal gland. Biochemical and Biophysical Research Communications.

[78] Doghman M., Delagrange P., Blondet A., Berthelon M.C., Durand P., Naville D. (2004). Agouti-related protein antagonizes glucocorticoid production induced through melanocortin 4 receptor activation in bovine adrenal cells: a possible autocrine control. Endocrinology.

[79] Bornstein S.R., Schuppenies A., Wong M.L., Licinio J. (2006). Approaching the shared biology of obesity and depression: the stress axis as the locus of gene-environment interactions. Molecular Psychiatry.

[80] de Kloet E.R., Sibug R.M., Helmerhorst F.M., Schmidt M.V. (2005). Stress, genes and the mechanism of programming the brain for later life. Neuroscience & Biobehavioral Reviews.

[81] Green J.G., McLaughlin K.A., Berglund P.A., Gruber M.J., Sampson N.A., Zaslavsky A.M. (2010). Childhood adversities and adult psychiatric disorders in the national comorbidity survey replication I: associations with first onset of DSM-IV disorders. Archives of General Psychiatry.

[82] Heim C., Nemeroff C.B. (2001). The role of childhood trauma in the neurobiology of mood and anxiety disorders: preclinical and clinical studies. Biological Psychiatry.

[83] Shonkoff J.P., Boyce W.T., McEwen B.S. (2009). Neuroscience, molecular biology, and the childhood roots of health disparities: building a new framework for health promotion and disease prevention. Journal of the American Medical Association.

