# Supplementary figures and images for: Insulin and obesity transform hypothalamic-pituitary-adrenal axis stemness and function in a hyperactive state
Source: Mol Metab. 2020 Nov 4;43:101112. doi: 10.1016/j.molmet.2020.101112 (PMC7691554; doi:10.1016/j.molmet.2020.101112)

## Slide 1
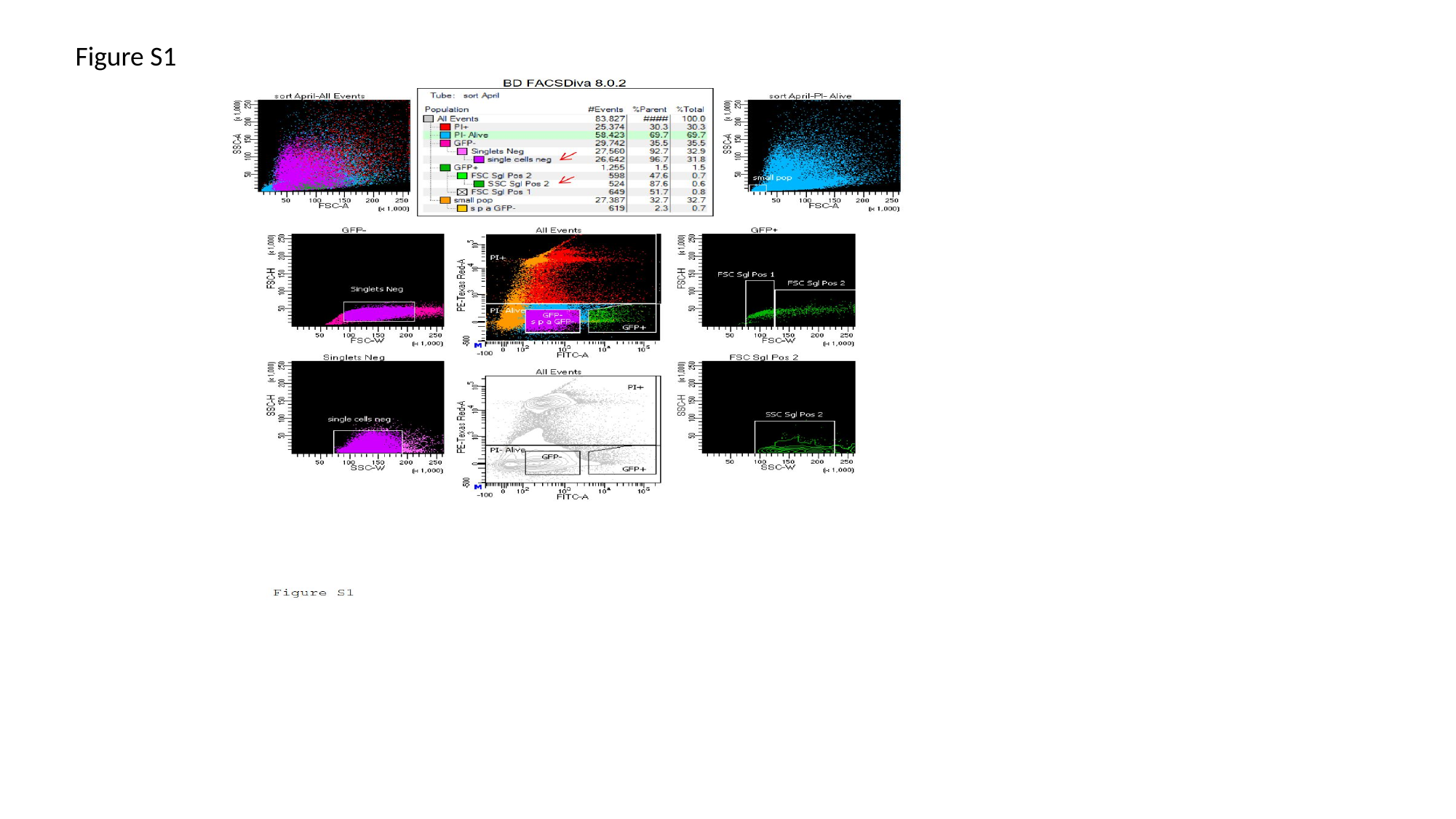

Figure S1

Supplement: Figure S1 — Gating strategy for fluorescent-activated cell sorting of pituitary stem cells. The anterior pituitary was isolated from Nestin-GFP mice and dissociated into a single-cell suspension. Living cells were identified as PI(−). GFP(+) and GFP(−) single cell populations were sorted (marked with red arrows). [file mmc1.pptx]

## Slide 1
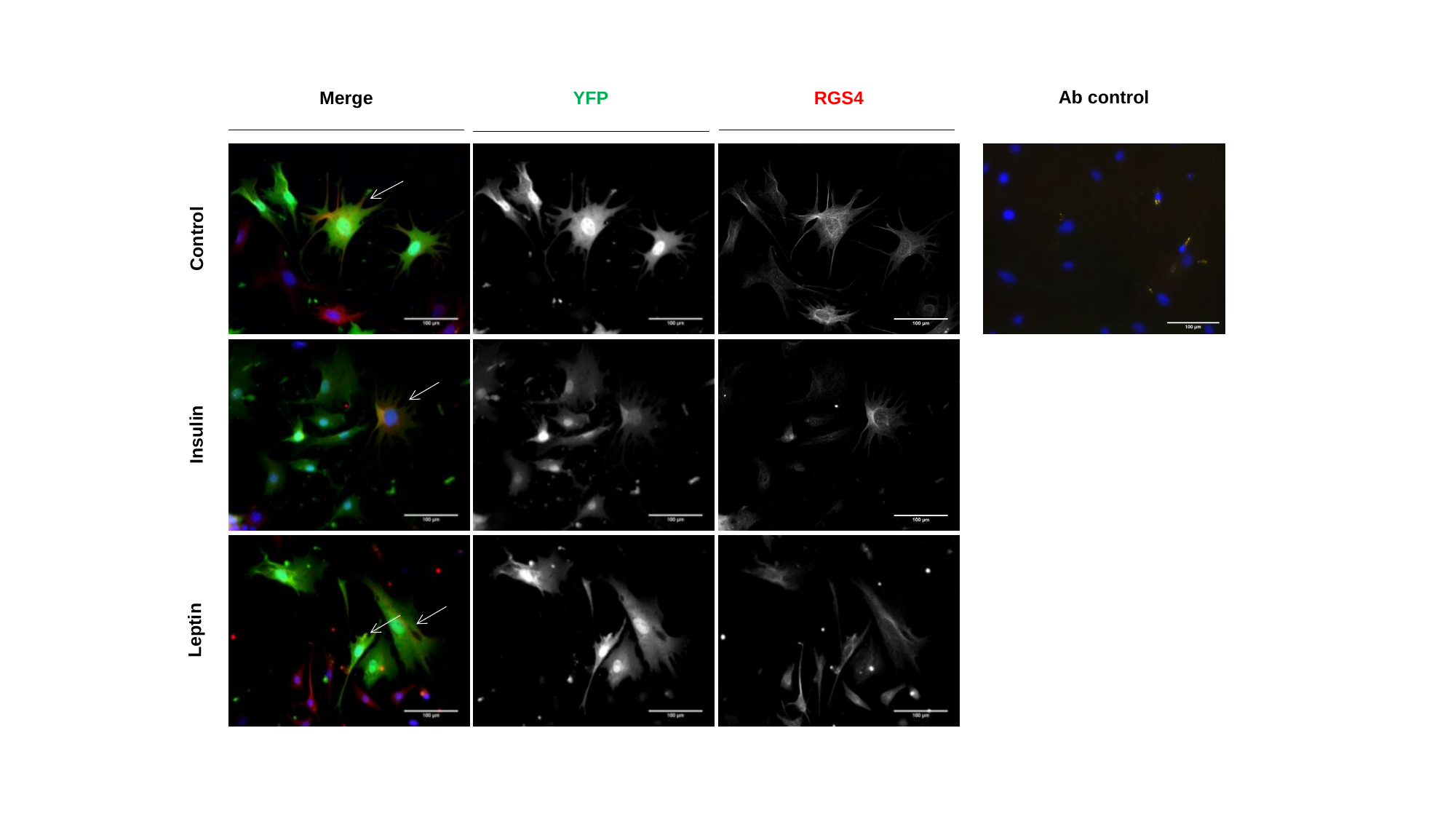

Ab control
Merge
YFP
RGS4
Control
Insulin
Leptin

Supplement: Figure S3 — In vitro lineage tracing of adrenocortical progenitors. Tracing of YFP(+) cells isolated from Nes-CreERT+/-;Rosa26-eYFP+/+ mice, in which recombination was induced in vitro. After differentiation for 7 days, the cells were immunostained for RGS4 marking zG cells and YFP marking Nestin-derived cells. Double-positive cells are indicated with arrows. Secondary antibodies alone were observed in Ab control. Representative images are shown. [file mmc3.pptx]

## Slide 1
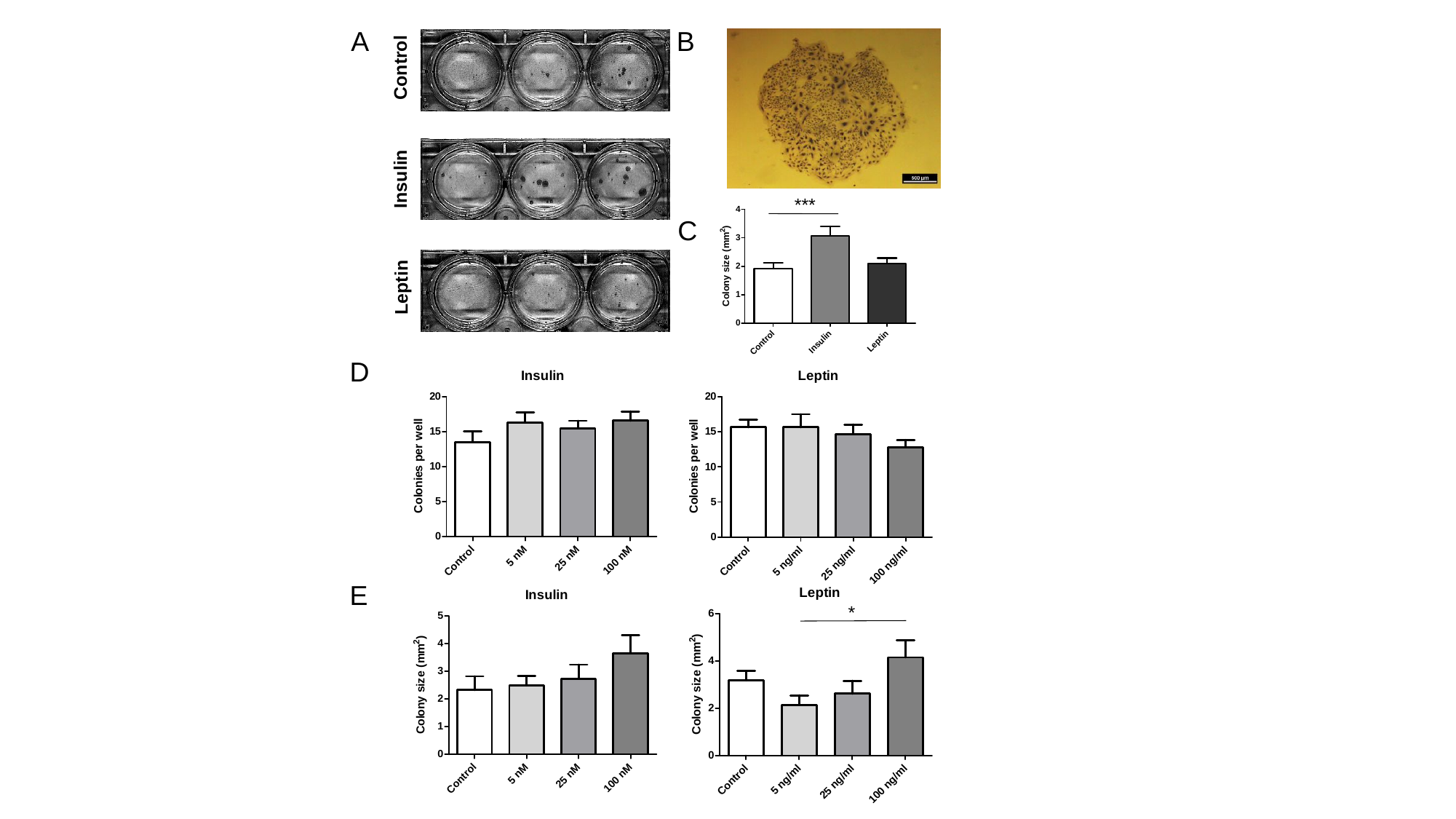

Control
A
B
Insulin
***
C
Leptin
D
E
*

Supplement: Figure S4 — In vitro culture of pituitary progenitors. (A) Cells from the pituitary anterior lobe of Nestin-GFP mice were isolated and cultured in stem cell supporting medium in the presence of insulin or leptin as indicated. (B) Single colony. (C) Average colony area. Data in C are presented as mean ± SEM (n = 3; biological replicates). Data were analyzed by one-way ANOVA and Bonferroni's post-test. ∗∗∗P < 0.001. (D) Cells from the pituitary anterior lobe of WT mice were isolated and cultured in stem cell supporting medium I containing different concentrations of insulin or leptin as indicated. The number of colonies per well was counted (n = 6; biological replicates). Data were analyzed by unpaired two-sided t-tests. (E) Average colony area (n = 3; biological replicates). Data were analyzed by unpaired two-sided t-tests. P = 0.0116 for leptin (100 ng/ml vs 5 ng/ml). [file mmc4.pptx]

## Slide 1
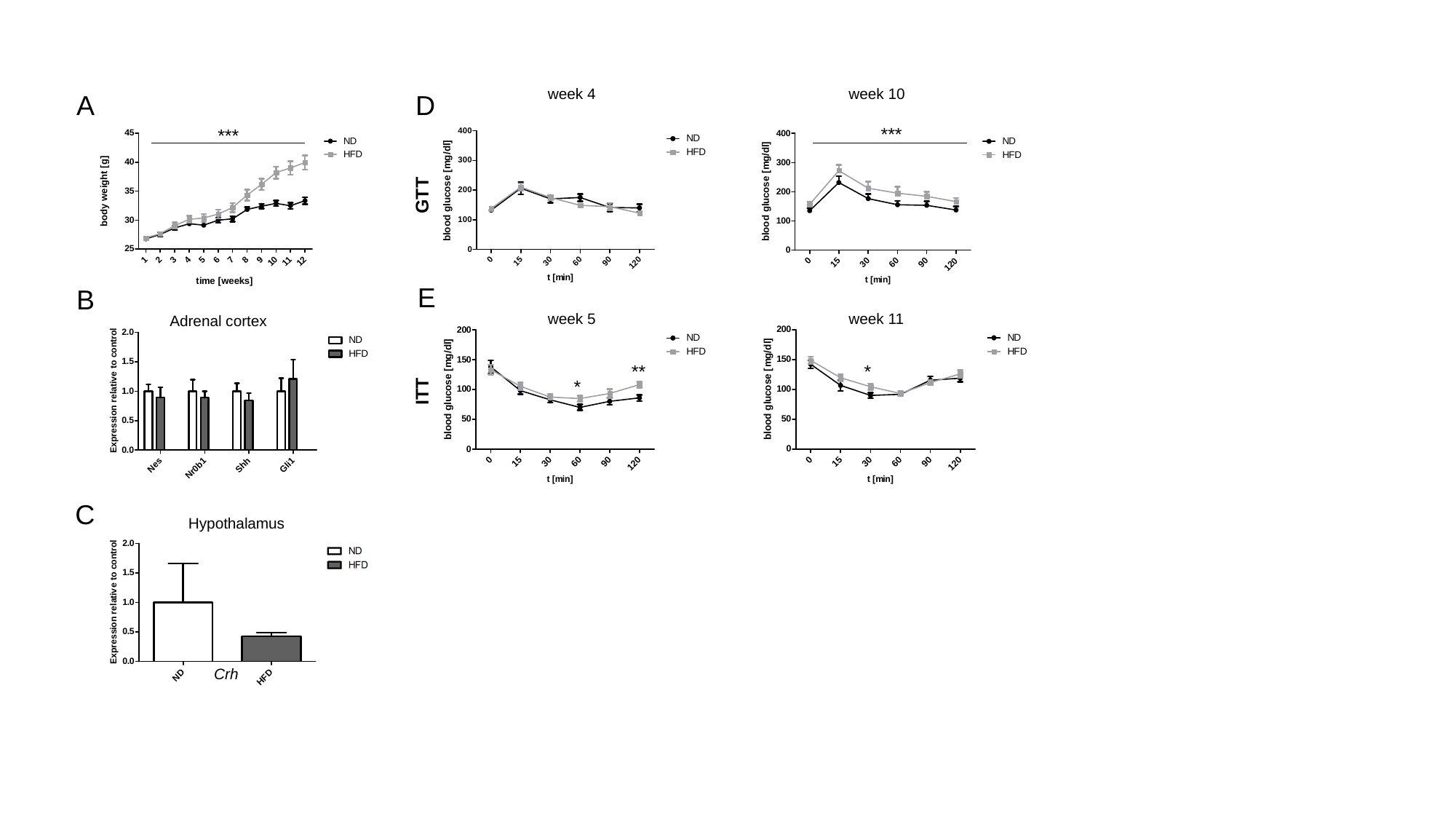

week 4
week 10
A
D
GTT
E
ITT
***
***
B
week 5
week 11
Adrenal cortex
**
*
*
C
Hypothalamus
Crh

Supplement: Figure S6 — HFD experiment. (A) Weight development of mice on 12 weeks of ND and HFD. Data were analyzed by two-way ANOVA and Bonferroni's post-test. (B) qRT-PCR analysis showing the relative expression of various stem cell/progenitor markers. (C) qRT-PCR analysis of the relative expression of Crh in the hypothalamus. (D) Glucose-tolerance tests were performed after 4 and 10 weeks. Data were analyzed by two-way ANOVA and Bonferroni's post-test. (E) Insulin-tolerance tests were performed after 5 and 11 weeks. P = 0.0480 and 0.0060 for 60 and 120 min for HFD vs ND for 5 weeks. P = 0.0431 for 30 min for HFD vs ND for 11 weeks. Data were analyzed by unpaired two-sided t-tests. Data are presented as mean ± SEM (n ≥ 6, biological replicates). ∗P < 0.05, ∗∗P < 0.01, and ∗∗∗P < 0.001. [file mmc6.pptx]
