# Supplementary material for: Insulin and obesity transform hypothalamic-pituitary-adrenal axis stemness and function in a hyperactive state
Source: Mol Metab. 2020 Nov 4;43:101112. doi: 10.1016/j.molmet.2020.101112 (PMC7691554; doi:10.1016/j.molmet.2020.101112)
Supplement: Figure S2 — In vitro cultures of adrenocortical progenitors. (A) Adrenocortical cells from Nestin-GFP mice were isolated and cultured under low-attachment conditions in the presence of insulin or leptin as indicated. (B) On day 7, the culture conditions were changed and differentiation was induced. Stimulation with insulin and leptin continued. The expression of GFP was tracked throughout the experiment and representative images are shown. [file mmc2.pptx]

## Slide 1
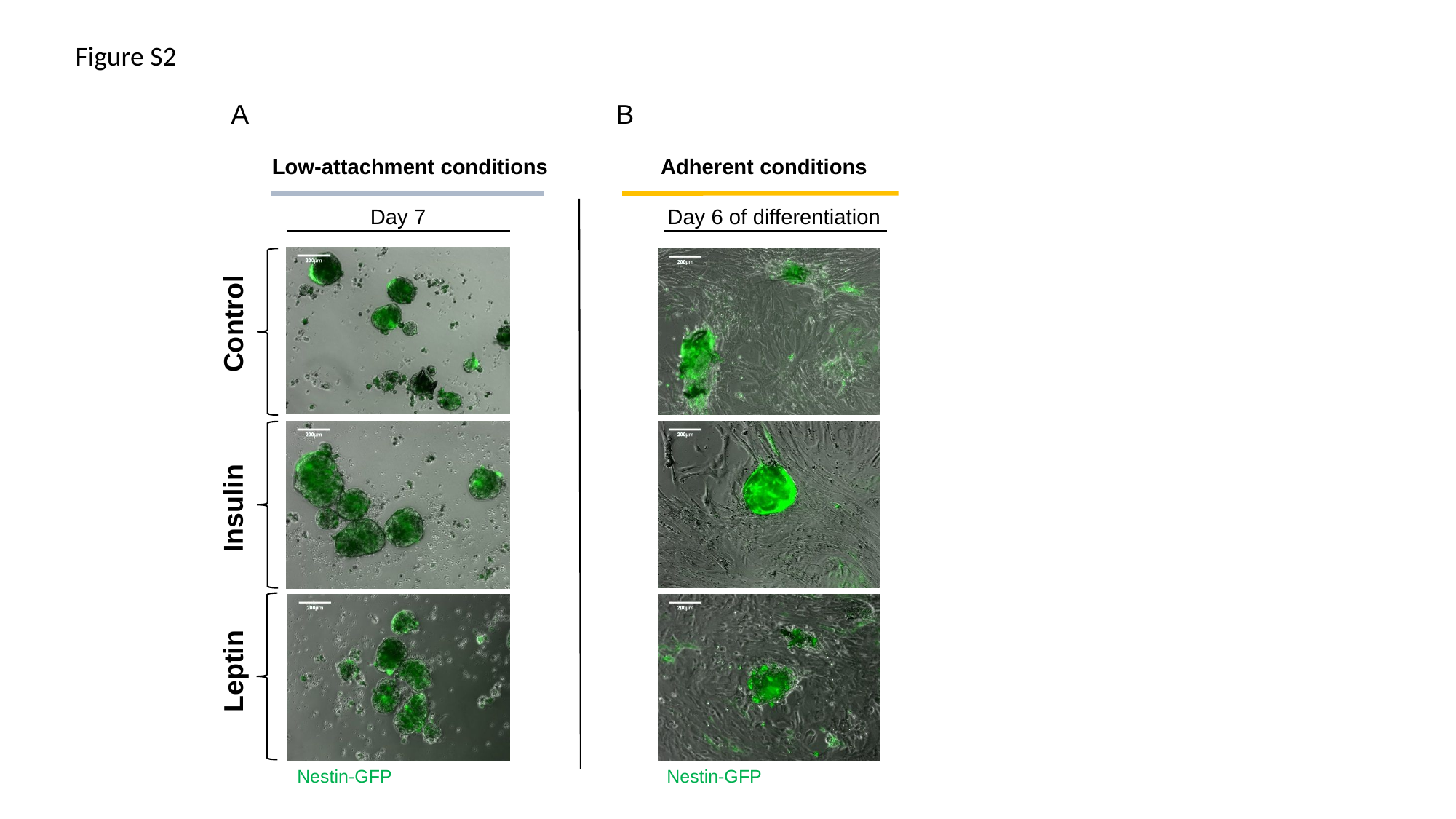

Figure S2
A
B
Low-attachment conditions
Adherent conditions
Day 6 of differentiation
Day 7
Control
Insulin
Leptin
Nestin-GFP
Nestin-GFP
