# Supplementary material for: Insulin and obesity transform hypothalamic-pituitary-adrenal axis stemness and function in a hyperactive state
Source: Mol Metab. 2020 Nov 4;43:101112. doi: 10.1016/j.molmet.2020.101112 (PMC7691554; doi:10.1016/j.molmet.2020.101112)
Supplement: Figure S5 — Antibody validation. (A) The antibody against CRHR1 was optimized and validated on pituitary stem cell colonies isolated from Nestin-GFP mice and cultured in vitro. Secondary antibodies alone were observed in Ab control. (B) Antibodies against StAR and Nestin were optimized and validated on adrenocortical progenitors, which were isolated from WT mice and differentiated into steroidogenic cells in vitro. Secondary antibodies alone were observed in Ab control. [file mmc5.pptx]

## Slide 1
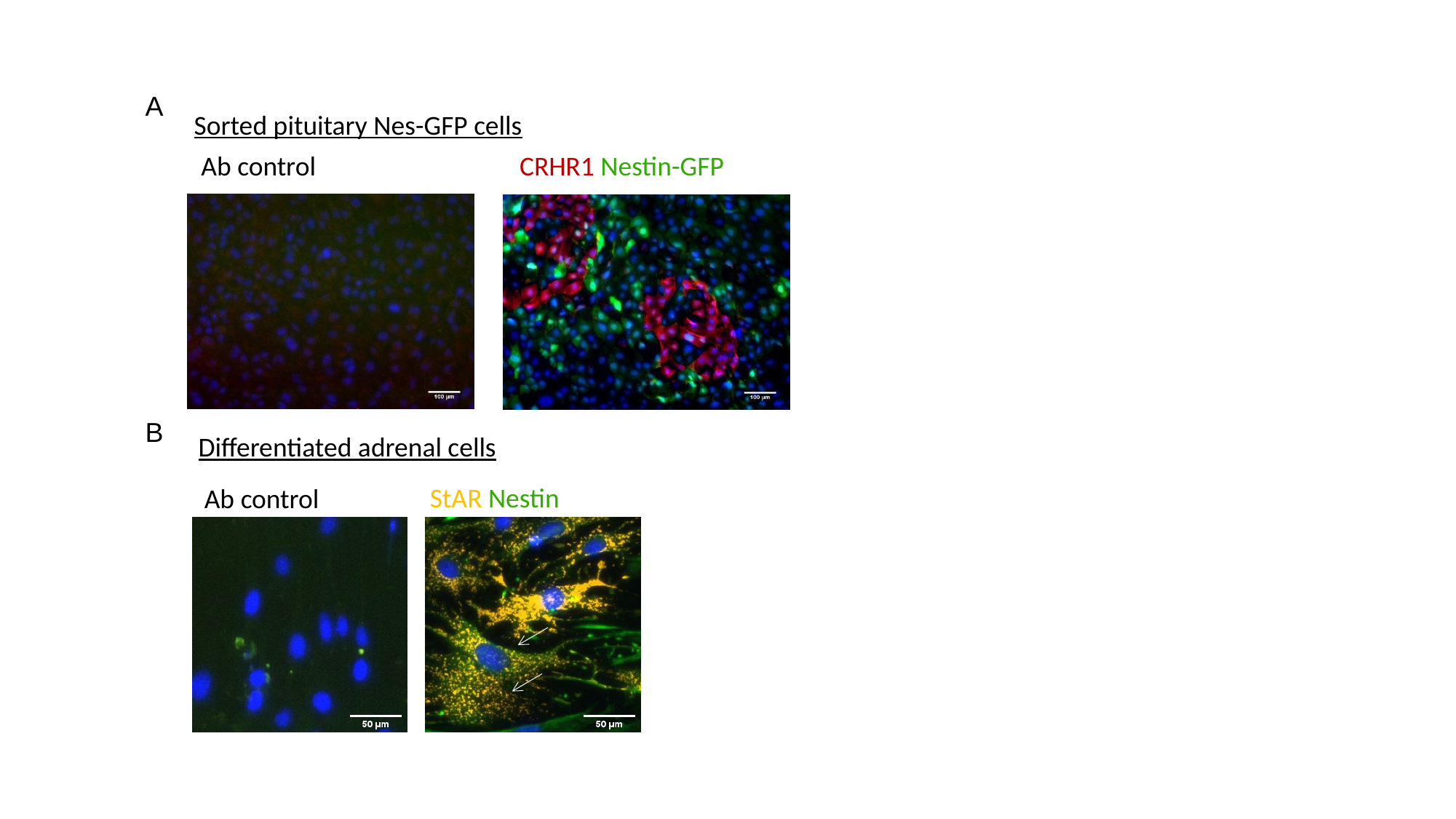

A
Sorted pituitary Nes-GFP cells
Ab control
CRHR1 Nestin-GFP
B
Differentiated adrenal cells
StAR Nestin
Ab control
