# Supplementary material for: Insulin and obesity transform hypothalamic-pituitary-adrenal axis stemness and function in a hyperactive state
Source: Mol Metab. 2020 Nov 4;43:101112. doi: 10.1016/j.molmet.2020.101112 (PMC7691554; doi:10.1016/j.molmet.2020.101112)
Supplement: Figure S7 — Eight-week-old ob/ob females and 10-month-old ob/ob males. (A) Final body weight of obese ob/ob mice and their controls. Data were analyzed by unpaired two-sided t-tests. (B) Representative images of immunostaining for zonation analysis of 8-week-old female ob/ob mice are shown. Scale bars, 200 μm. (C) Nestin(+) cells migrated and differentiated into StAR(+) cells. Arrow indicates double-positive cell for Nestin and StAR. Scale bar, 20 μm. Representative images are shown. ∗∗∗P < 0.001. [file mmc7.pptx]

## Slide 1
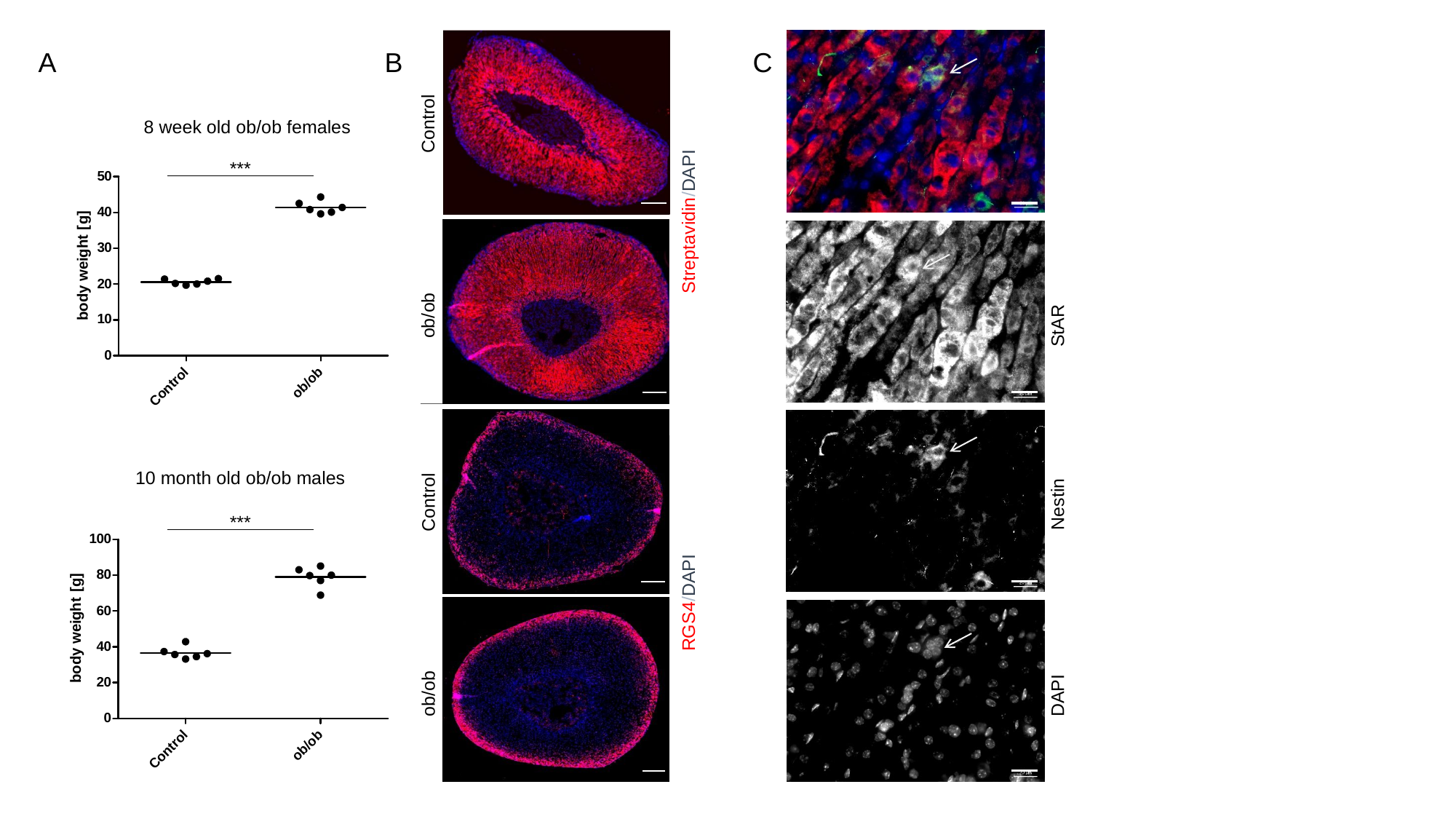

A
B
C
Control
8 week old ob/ob females
***
Streptavidin/DAPI
ob/ob
StAR
10 month old ob/ob males
Nestin
Control
***
RGS4/DAPI
DAPI
ob/ob
