# Supplementary material for: Insulin and obesity transform hypothalamic-pituitary-adrenal axis stemness and function in a hyperactive state
Source: Mol Metab. 2020 Nov 4;43:101112. doi: 10.1016/j.molmet.2020.101112 (PMC7691554; doi:10.1016/j.molmet.2020.101112)
Supplement: Supplementary file 8 [file mmc8.docx]

**Table S1. List of antibodies**

| Antibody | SOURCE | Identifier |
| --- | --- | --- |
| Chicken polyclonal anti-GFP | Abcam | Cat# ab13970; RRID:AB_300798 |
| Rabbit polyclonal anti-RGS4 | Thermo Fisher | Cat# PA5-22332  RRID:AB_11155787 |
| Rabbit-polyclonal anti-Sox2 | Abcam | Cat# ab97959  RRID:AB_2341193 |
| Goat-polyclonal anti-CRHR1 | Novus Biologicals | Cat# NBP1-00175  RRID:AB_1502951 |
| Rabbit-polyclonal anti-Ki67 | Abcam | Cat# ab15580  RRID:AB_443209 |
| Chicken polyclonal anti-Nestin | Novus Biologicals | Cat# NB100-1604  RRID:AB_2282642 |
| Rabbit polyclonal anti-StAR, clone FL-285 | Santa Cruz Biotechnology | Cat# sc-25806; RRID:AB_2115937 |
| Streptavidin, Alexa Fluor 555 conjugate | Thermo Fisher | Cat# S21381  RRID:AB_2307336 |
| Cy3-goat-anti-rabbit | Jackson ImmunoResearch | Cat# 111-165-144; RRID:AB_2338006 |
| Cy3-donkey-anti-goat | Jackson ImmunoResearch | Cat# 705-165-147; RRID:AB_2307351 |
| Alexa Fluor 488-donkey-anti-chicken | Jackson ImmunoResearch | Cat# 703-546-155; RRID:AB_2340376 |
