# Supplementary material for: Insulin and obesity transform hypothalamic-pituitary-adrenal axis stemness and function in a hyperactive state
Source: Mol Metab. 2020 Nov 4;43:101112. doi: 10.1016/j.molmet.2020.101112 (PMC7691554; doi:10.1016/j.molmet.2020.101112)
Supplement: Supplementary file 9 [file mmc9.docx]

**Table S2. List of primers and amplification conditions for RT-PCR**

| Gene | Primer sequence | Product size (base pairs) |
| --- | --- | --- |
| *Actb* | F: GAGCACAGCTTCTTTGCAGCTCCTT | 280 |
|  | R: TGCCATGTTCAATGGGGTACTTCAG |  |
| *Nes* | F: CTCTGCTGGAGGCTGAGAAC | 176 |
|  | R: ATTAGGCAAGGGGGAAGAGA |  |
| *Gli1* | F: TGGATCGGATGGGAGGTCTT | 298 |
|  | R: ACCTCTGGCTCCTCCTGTAG |  |
| *Shh* | F: TGAACGGACCTTCAAGAGCC | 190 |
|  | R:GCAGGAGCATAGCAGGAGAG |  |
| *Nr0b1* (*Dax1*) | F: ATGGAGATCCCGGAGACCAA | 236 |
|  | R: AAGAGCACGGTCCCTTTCAG |  |
| *Mc2r* | F: CAAAGCCAAGGAGAGGAGCATTATT | 149 |
|  | R: GGTGTTTGCCGTTGACTTACAGAAA |  |
| *Cyp11a1* | F: TGGGTGGCCTATCACCAGTATTATC | 103 |
|  | R: CCATCACCTCTTGGTTTAGGACGAT |  |
| *Actb* TaqMan | F: TATTGGCAACGAGCGGTT | 75 |
|  | R: ATGCCACAGGATTCCATACCC |  |
| *Actb* TaqMan probe | CCTGAGGCTCTTTTTCCAGCCTTCCTTCT |  |
| *Cyp11b1* TaqMan | F: AGAGCTGGCAGAGGGTCGT | 79 |
|  | R: TGGCATCCATTGACAGAGTTCT |  |
| *Cyp11b1* TaqMan probe | CACAGTCCTGGAGTGTCACAGCAGAGCT |  |
| *Cyp11b2* TaqMan | F: CAGACTCGGCAGCTCTCAGA | 77 |
|  | R: ATGGCGTCGAGAGGCAAA |  |
| *Cyp11b2* TaqMan probe | CTACAGTGGCATTGTGGCGGAACTAATATCTCA |  |
| *Star* | F: CTGGCTGGAAGTCCCTCCAAGACTA | 224 |
|  | R: AGGCTTCCTGTGAGAGCTTCCAATG |  |
| *Nr5a1* | F: CTTTAAGGAGCTGGAGGTGGCT | 114 |
|  | R: ATGCTGTCTTCCTTGCCGTACT |  |
| *RGS4* | F: ACATCGGCTGGGGTTCCTGC | 166 |
|  | R: AGCTGCCAGTCCACATTCATGGT |  |
| *Sox2* | F: TCGGTGATGCCGACTAGAAAA | 177 |
|  | R: GCGCCTAACGTACCACTAGAACTT |  |
| *Tbx19* | F: TTTATCTTGGCCACGCTTAGG | 76 |
|  | R: CCCAGAACGGCTTGAGAGTAA |  |
| *Pouf1f1* | F: CCACAGCGACAGGACTTCAT | 203 |
|  | R: CCGCCTGAGTTCCTGCTTAA |  |
| *Pomc* | F: GCGGGAGAGAAAGCCGAGTCA | 156 |
|  | R: AGGGACCCCGTCCTGTCCTA |  |
| *Crhr1* | F: AGGGCTTCTTCGTGTCTGTG | 237 |
|  | R: TTGCATCATTTCCCCAGCCT |  |
| *Crh* | F: GAGAAGAGAGCGCCCCTAAC | 236 |
|  | R: TCTGTTGAGATTCCCCAGGC |  |
| *Npy* | F: CGCCCCCAGAACAAGGCTTGAA | 166 |
|  | R: GGGAAATGGGGCGGAGTCCA |  |
| *Agrp* | F: CTGACTGCAATGTTGCTGAG | 406 |
|  | R: CAACATCCATTGGCTAGGTG |  |

F: forward primer, R: reverse primer.
